# Treatment of carotid stenosis: an updated review

**DOI:** 10.3389/fstro.2026.1849877

**Published:** 2026-06-24

**Authors:** Antônio Vinícius Pimentel Lima, Maria Victória Pimentel Lima, Marina Trombin Marques, Vivian Dias Batista Gagliardi, Rubens Jose Gagliardi

**Affiliations:** 1 Discipline of Neurology, Department of Medicine, Irmandade da Santa Casa de Misericórdia de São Paulo, São Paulo, Brazil; 2 School of Medicine, Tiradentes University, Aracaju, Brazil; 3 Department of Neurology and Neurosurgery, Paulista School of Medicine, Federal University of São Paulo, São Paulo, Brazil; 4 Discipline of Neurology, Department of Internal Medicine, School of Medical Sciences of Santa Casa de São Paulo, São Paulo, Brazil

**Keywords:** angioplasty, atherosclerosis, carotid endarterectomy, carotid stenosis, stroke

## Abstract

**Introduction:**

Atherosclerotic carotid stenosis represents one of the main treatable causes of ischemic stroke, accounting for approximately 10%−15% of all cerebrovascular events. Treatment of this condition has evolved significantly over the past decades, shifting from exclusively surgical approaches to a paradigm that includes optimized medical treatment, carotid endarterectomy, and stenting. Recent 2025 publications, including CREST-2 and ECST-2, have brought paradigm-shifting data that challenge previously established concepts.

**Objective:**

To provide an extensive and rigorous narrative review on the treatment of carotid stenosis, including common and internal carotid arteries.

**Methods:**

This narrative review was carried out using the PubMed/MEDLINE, LILACS-VHL, Google Scholar, and SciELO databases. DeCS and MeSH descriptors were employed to identify articles published from 2020 to 2025, without restrictions regarding language or geography.

**Results:**

In the CREST-2 stenting trial, adding carotid stenting to optimal medical therapy (OMT) was superior to OMT alone for the 4-year composite primary endpoint (any periprocedural stroke or death, or subsequent ipsilateral ischemic stroke): 2.8% vs. 6.0% (*p* = 0.02; number needed to treat = 31). This net benefit was driven by a lower rate of ipsilateral stroke beyond the periprocedural window and was obtained despite a higher upfront periprocedural risk in the stenting arm. In the parallel endarterectomy trial, adding endarterectomy to OMT did not reach statistical significance (3.7% vs. 5.3%; p = 0.24). ECST-2, in its 2-year interim analysis of asymptomatic or low-to-intermediate-risk symptomatic patients, showed no benefit of revascularization added to OMT.

**Conclusion:**

Contemporary data indicate a paradigm shift in the management of carotid stenosis. Optimal medical therapy (OMT), intensified by increasingly stringent LDL-c and blood-pressure targets, is the cornerstone of management for all patients and the principal driver of the marked decline in stroke risk observed over the past two decades. Against this strengthened medical background, the role of revascularization has become more selective: in high-grade asymptomatic stenosis, adding carotid stenting to OMT conferred a modest absolute benefit over OMT alone, whereas carotid endarterectomy (the long-standing gold standard) did not show a statistically significant benefit. These findings support an individualized, plaque- and risk-based strategy in which OMT is universal and revascularization (preferentially by stenting when an intervention is chosen) is reserved for selected patients, with timing and modality guided by stenosis severity, plaque vulnerability, life expectancy, and patient preference.

## 1 Introduction

### 1.1 Definition and fundamental concepts

Atherosclerotic carotid stenosis is defined as the progressive narrowing of the arterial lumen resulting from the accumulation of atheromatous plaques within the walls of the carotid arteries, predominantly at the carotid bifurcation and the proximal segment of the internal carotid artery. This condition constitutes one of the leading modifiable causes of ischemic stroke, accounting for 10%−15% of all cerebrovascular events (Sacco et al., 2013). The *Global Stroke Fact Sheet 2025* of the *World Stroke Organization* (WSO), published by Feigin et al. (2025) estimates that the global cost of stroke exceeds 890 billion dollars, representing 0.66% of the Gross Domestic Product (GDP) worldwide.

The magnitude of this burden is underscored by the most recent data from the *Global Burden of Disease 2021* (Feigin et al., 2025). From 1990 to 2021, there was a substantial increase in the global burden of stroke: a 70% rise in incident cases, 44% in deaths, 86% in prevalence, and 32% in disability-adjusted life years (Feigin et al., 2025). Approximately 87% of stroke-related deaths and 89% of DALYs are concentrated in low- and middle-income countries, highlighting the relevance of primary and secondary prevention strategies in these settings (Feigin et al., 2025).

From a pathophysiological standpoint, carotid atherosclerotic disease shares common mechanisms with atherosclerosis in other vascular territories; however, it exhibits distinct features that influence both the risk of events and the therapeutic approach (Depuydt et al., 2020). A recent estimate indicates that approximately 816 million individuals aged 30–79 years harbor some degree of carotid plaque worldwide, of whom 137.6 million reside in Europe, while approximately 58 million individuals present with significant carotid stenosis (1.5% of the population) (Musialek et al., 2025).

### 1.2 Anatomy of the carotid arteries

The carotid system comprises the right and left common carotid arteries, which arise asymmetrically: the right common carotid artery originates from the brachiocephalic trunk, whereas the left arises directly from the aortic arch. Each common carotid artery ascends through the neck without giving off significant branches until its bifurcation, typically located at the level of the fourth cervical vertebra, where it divides into the internal and external carotid arteries (Musialek et al., 2025).

This aortic arch configuration is subject to anatomical variation in approximately 13%−27% of the general population. The most prevalent variant is the so-called bovine arch, in which the left common carotid artery shares a common origin with the brachiocephalic trunk or arises directly from it; less frequently, a separate vertebral or thyroid trunk may arise from the arch. These variants are clinically relevant for endovascular procedures, since they directly modify the catheter trajectory through the aortic arch and may increase the technical difficulty of transfemoral carotid stenting. Aortic arch elongation and atheromatous burden, more prevalent in older patients, are recognized contributors to the higher periprocedural embolic risk observed in this population during transfemoral CAS, a finding that has informed the development and progressive adoption of transcarotid artery revascularization (TCAR), which bypasses the aortic arch entirely (Hicks et al., 2026; Voeks et al., 2011).

The internal carotid artery constitutes the primary arterial supply to the anterior and middle cerebral hemispheres. After its origin at the bifurcation, it ascends through the neck (cervical segment), enters the skull through the carotid canal (petrous segment), traverses the cavernous sinus (cavernous segment), and terminates in the middle cranial fossa, where it divides into its major intracranial branches: the middle cerebral artery, anterior cerebral artery, ophthalmic artery, posterior communicating artery, and anterior choroidal artery (Musialek et al., 2025).

The carotid bulb, a region of physiological dilation located at the origin of the internal carotid artery, represents the preferential site for the development of atherosclerotic plaques owing to local hemodynamic characteristics, including turbulent flow, low shear stress, and oscillation of the wall shear stress vector. These conditions promote endothelial dysfunction, lipid accumulation, and the progression of atherosclerosis (Musialek et al., 2025).

### 1.3 Epidemiology

The prevalence of carotid stenosis increases progressively with age. Epidemiological data from four population-based studies demonstrate that the prevalence of stenosis greater than 50% ranges from 0.2% to 3.1% in women and from 0.1% to 3.1% in men, whereas stenosis greater than 70% has a prevalence of 0.1% to 1.7% in women and 0.1% to 2.3% in men, depending on the age group assessed (Naylor et al., 2023).

Carotid atherosclerotic disease is closely associated with traditional cardiovascular risks factors, including hypertension, diabetes mellitus, dyslipidemia, smoking, and advanced age. Patients with atherosclerosis in other vascular territories have an increased risk of concomitant carotid disease: the prevalence of carotid stenosis is 5%−9% in patients with coronary artery disease and 14%−19% in patients with peripheral arterial disease (Musialek et al., 2025).

In the international REACH registry (*Reduction of Atherothrombosis for Continued Health*), which enrolled 33,493 outpatients with risk factors or established atherosclerotic disease, those with asymptomatic carotid stenosis greater than 70% exhibited significantly higher rates of transient ischemic attack (3.51% vs. 1.61%; *p* < 0.0001), non-fatal stroke (2.65% vs. 1.75%; *p* = 0.0009), and fatal stroke (0.49% vs. 0.26%; *p* = 0.04) compared with the remaining participants (Naylor et al., 2023).

### 1.4 Pathophysiology of carotid stenosis

The pathophysiology of carotid atherosclerotic disease involves complex mechanisms including endothelial dysfunction, accumulation of modified lipoproteins in the arterial intima, recruitment and activation of inflammatory cells, foam cell formation, smooth muscle cell proliferation, and, ultimately, the development of vulnerable plaques at risk of rupture and thrombosis. Single-cell RNA transcriptomic studies have enabled the identification of specific components of carotid plaques and their molecular characteristics (Depuydt et al., 2020).

Although the mechanisms of atherosclerosis are similar across different vascular territories, accumulating evidence suggests that carotid atherosclerotic plaques may exhibit distinct characteristics that require specific therapeutic approaches. Compared with coronary plaques, where fibrous cap rupture typically occurs when its thickness is less than 65 micrometers, in vulnerable carotid lesions, rupture may occur at a significantly greater cap thickness, approximately 200 micrometers (Feigin et al., 2025; Depuydt et al., 2020).

The mechanisms by which carotid stenosis causes cerebrovascular events predominantly involve artery-to-artery embolization, in which plaque fragments or thrombi formed on its surface dislodge and occlude distal cerebral arteries. This atheroembolic mechanism accounts for the majority of ischemic events related to carotid disease. Less frequently, high-grade stenoses or occlusions may cause ischemia through a hemodynamic mechanism, when the compensatory capacity through the circle of Willis and other anastomotic circuits is insufficient.

The characterization of carotid plaque morphology and composition has gained increasing importance in risk stratification. Features associated with greater vulnerability include: increased plaque volume, reduced echogenicity on ultrasound, presence of a large lipid-rich necrotic core, intraplaque hemorrhage, thin or ruptured fibrous cap, ulcerations, neovascularization, and active inflammation. A meta-analysis of 42 articles on fundamental characteristics of carotid plaques revealed that men more frequently present with larger, calcified plaques with lipid-rich necrotic cores and intraplaque hemorrhage compared with women (Van Dam-Nolen et al., 2023).

### 1.5 Clinical presentation

Carotid stenosis may be classified as asymptomatic or symptomatic, a distinction that is fundamental for therapeutic decision-making. The most commonly used definition considers stenosis as asymptomatic when it has never caused neurological symptoms or is not associated with recent neurological events, typically within the preceding 6 months, although cutoff points range from 1 to 12 months or may even include remote symptoms (Naylor et al., 2023).

Most symptoms caused by carotid atherosclerotic disease result from plaque inflammation and rupture with subsequent embolization of thrombus or debris, leading to occlusion of retinal or cerebral arteries (Naylor et al., 2023). These occlusions cause ischemia responsible for focal neurological deficits, which are defined as ischemic stroke when there is anatomopathological or imaging of evidence of focal cerebral or retinal ischemic injury in a recognized vascular distribution, even if the clinical deficit presented is transient (Sacco et al., 2013). It is defined as TIA when it produces a transient clinical deficit without radiological or anatomopathological findings (Easton et al., 2009).

Focal neurological symptoms include, alone or in combination: motor deficits (such as isolated paresis of the hand, arm, face, or less commonly, the lower extremity), sensory deficits, aphasia (when the left hemisphere is affected), hemispatial neglect (predominantly with right hemisphere involvement), and hemianopia. Ocular manifestations related to carotid stenosis include transient monocular visual loss (*amaurosis fugax*), retinal artery occlusion, and chronic ocular ischemic syndrome (Musialek et al., 2025).

Patients with TIA caused by carotid stenosis carry a markedly increased risk of stroke, which may reach 20% within the first 3 months in studies conducted two decades ago, although more recent registries indicate a risk of approximately 6% in the first year. Carotid stenosis greater than 50% remains the most robust predictor of a new vascular event following TIA (Easton et al., 2009).

The ABCD (Feigin et al., 2025) score remains a widely employed clinical instrument for early stratification of stroke risk following TIA. It is composed of five clinical parameters—*Age* (≥60 years; one point), *Blood pressure* (systolic ≥ 140 mmHg or diastolic ≥ 90 mmHg at first assessment; one point), *Clinical features* of TIA (unilateral weakness: two points; speech disturbance without weakness: one point), *Duration* of symptoms (≥60 min: two points; 10–59 min: one point), and *Diabetes* (one point)—yielding a total score ranging from 0 to 7 and stratifying patients into low (0 to 3), moderate (4 to 5), and high (6 to 7) risk categories. In the original derivation and validation cohorts, the 2-day stroke risks were 1.0%, 4.1%, and 8.1% for low, moderate, and high-risk patients, respectively, with corresponding 7-day risks of 1.2%, 5.9%, and 11.7%, and 90-day cumulative risks approaching 17.8% in the high-risk stratum (Naylor et al., 2023). Importantly, in patients in whom carotid atherosclerotic disease is the underlying mechanism of TIA, the ABCD (Feigin et al., 2025) score should be interpreted in conjunction with vascular imaging findings, since the documented presence of an ipsilateral carotid stenosis ≥50% *per se* confers high-risk status independent of the clinical score and justifies expedited carotid evaluation and revascularization within the first 14 days following the index event, when revascularization is indicated (Naylor et al., 2023; Bonati et al., 2021; Paraskevas and AbuRahma, 2024).

### 1.6 Diagnostic imaging

Carotid duplex ultrasonography with Doppler represents the first-line examination for the identification and initial assessment of carotid stenosis owing to its availability, absence of ionizing radiation, low cost, and ability to provide both anatomical and functional information. However, its accuracy in the precise determination of moderate-to-severe stenosis grades is limited. In 2004, an analysis of 5,893 Doppler records from 338 accredited laboratories demonstrated significant variability in stenosis classification across different centers (Rothwell et al., 2004).

A precise quantification of carotid stenosis severity is critical for therapeutic decision-making, and two distinct angiographic methods, established by the landmark trials of the early 1990s, remain in clinical use. The NASCET method calculates the percentage of stenosis by comparing the residual luminal diameter at the site of maximal narrowing with the diameter of the normal distal internal carotid artery beyond the stenosis. The ECST method, in contrast, compares the residual luminal diameter at the stenosis with the estimated original outer diameter of the artery at the same level. As a consequence, both methods yield substantially different numerical estimates for the same anatomical lesion: approximately 50% NASCET corresponds to 70% ECST; 70% NASCET corresponds to approximately 82% ECST; and 80% NASCET corresponds to approximately 88% ECST (North American Symptomatic Carotid Endarterectomy Trial Collaborators, 1991; Warlow, 1991; Bierens et al., 2025; Albricker et al., 2023). Contemporary clinical trials and international guidelines have progressively standardized the use of the NASCET method for reporting stenosis severity, although the ECST measurement is occasionally retained when interpreting older datasets.

The multiparametric ultrasonographic criteria adopted by the 2023 DIC-CBR-SBACV consensus, together with the corresponding NASCET and ECST percentage equivalents and the hemodynamic thresholds applied in the CREST-2 trial, are summarized in Table 1.

**Table 1:** Multiparametric ultrasonographic criteria for the classification of internal carotid artery stenosis severity, according to the DIC-CBR-SBACV 2023 update (Albricker et al., 2023), with corresponding NASCET and ECST stenosis grades (Bierens et al., 2025; Sacco et al., 2013; Easton et al., 2009).

Stenosis severity (NASCET^*^)	Corresponding ECST^†^ grade	PSV^‡^ (cm/s)	EDV^§^(cm/s)	PSV ICA^¶^/CCA^**^ratio	EDV ICA^¶^/CCA^**^ratio
<50%	<70%	<140	<40	<2.0	<2.6
50%−69%	70%−82%	140–230	40–100	2.0–4.0	2.6–5.5
70%−99%	82%−99%	>230	>100	>4.0	>5.5
>90%	>94%	>400	>140	>5.0	>5.5
Sub-occlusion	Sub-occlusion	Variable	Variable	Filiform flow	Variable
Occlusion	Occlusion	Absent	Absent	Not applicable	Not applicable

Source: Albricker et al. (2023), Creative Commons Attribution 4.0 International License (CC BY 4.0).

Source: Adapted with permission from Albricker et al. (2023), with NASCET-ECST correlation derived from Rothwell et al. (2003) and Bierens et al. (2025).

The CREST-2 trial adopted hemodynamic thresholds equivalent to the 70%−99% NASCET category (Brott et al., 2026). The NASCET method uses the diameter of the normal distal ICA as the denominator, whereas the ECST method uses the estimated original outer diameter of the vessel at the site of the stenosis, which systematically yields higher percentage values for the same anatomical lesion. The hemodynamic thresholds adopted by CREST-2 (PSV ≥ 230 cm/s + EDV ≥ 100 cm/s or PSV ICA/CCA ratio ≥ 4.0) fall within the 70%−99% NASCET category (≈82%−99% ECST) (Brott et al., 2026). ^*^NASCET, North American Symptomatic Carotid Endarterectomy Trial; ^†^ECST, European Carotid Surgery Trial; ^‡^PSV, peak systolic velocity; ^§^EDV, end-diastolic velocity; ^¶^ICA, internal carotid artery; ^**^CCA, common carotid artery.

From an ultrasonographic standpoint, the 2023 systematic recommendation of the Department of Cardiovascular Imaging of the Brazilian Society of Cardiology, jointly endorsed by the Brazilian College of Radiology and the Brazilian Society of Angiology and Vascular Surgery, advocates a multiparametric approach that integrates peak systolic velocity (PSV), end-diastolic velocity (EDV), the PSV ratio between the internal carotid artery (ICA) and the common carotid artery (CCA), and the EDV ICA/CCA ratio, alongside the bidimensional and Doppler-color anatomical evaluation of the plaque (Albricker et al., 2023). Stenoses below 50% are typically associated with PSV < 140 cm/s, EDV < 40 cm/s, and PSV ICA/CCA ratio < 2.0; stenoses of 50%−69%, with PSV between 140 and 230 cm/s, EDV between 40 and 100 cm/s, and PSV ratio between 2.0 and 4.0; stenoses of 70%−99%, with PSV > 230 cm/s, EDV >100 cm/s, and PSV ratio >4.0; and stenoses >90% frequently demonstrate PSV > 400 cm/s and PSV ratio > 5.0 (Rothwell et al., 2004; Albricker et al., 2023). The CREST-2 trial adopted threshold criteria largely aligned with these consensus references (PSV ≥ 230 cm/s combined with EDV ≥ 100 cm/s or a PSV ICA/CCA ratio ≥ 4.0) to confirm eligibility of severe asymptomatic stenoses (≥70%), reinforcing the contemporary applicability of multiparametric duplex criteria (Albricker et al., 2023; Gornik et al., 2021).

Notably, the combination of plaque-based and hemodynamic ultrasonographic findings substantially improves stroke risk stratification: in a meta-analysis of asymptomatic patients deposited in the American Dryad data repository, the simultaneous presence of carotid plaque echolucency and ≥1 spontaneous microembolic signal on transcranial Doppler conferred an odds ratio (OR) of 10.61 (95% CI, 2.98–37.82; *p* = 0.0003) for ipsilateral ischemic events, substantially exceeding the predictive performance of stenosis severity alone (Albricker et al., 2023; Fabiani et al., 2020).

Beyond ultrasound, computed tomography angiography (CTA) provides high spatial resolution for direct measurement of the residual luminal diameter, which correlates linearly with the angiographic estimate of stenosis severity. A residual lumen diameter ≤ 1.3 mm typically corresponds to stenoses ≥ 70% by the NASCET method, with reported sensitivity of 88.2%, specificity of 92.4%, and negative predictive value of 98%. CTA further enables the identification of carotid sub-occlusion, a clinically distinct entity characterized by reduced caliber of the affected distal ICA, in which an affected-to-contralateral ICA ratio < 0.87 or an affected ICA-to-ipsilateral external carotid artery ratio < 1.27 are highly suggestive findings (Albricker et al., 2023). Magnetic resonance angiography (MRA), particularly contrast-enhanced and time-of-flight sequences, provides comparable diagnostic accuracy and offers the unique additional benefit of vessel-wall imaging, which permits direct visualization of plaque vulnerability features such as intraplaque hemorrhage, lipid-rich necrotic core, and fibrous cap integrity, features increasingly recognized as superior predictors of stroke recurrence to stenosis severity alone (Musialek et al., 2025; Albricker et al., 2023; Brott et al., 2026).

**Figure 1:**
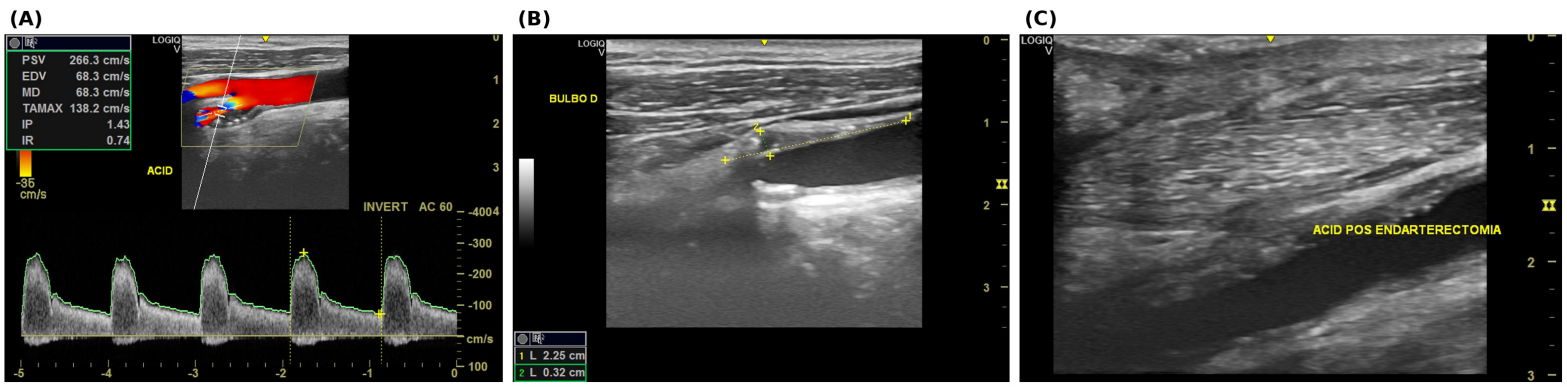
Carotid duplex ultrasonography of a patient with 70%−99% stenosis (NASCET) of the right internal carotid artery (RICA). **(A)** Color Doppler and spectral analysis of the RICA demonstrating an elevated peak systolic velocity (PSV) of 266.3 cm/s and end-diastolic velocity (EDV) of 68.3 cm/s, with PSV exceeding the CREST-2 cut-off (≥230 cm/s) for high-grade stenosis. **(B)** B-mode ultrasonography of the right carotid bulb (BULBO D) prior to carotid endarterectomy (CEA), showing heterogeneous atherosclerotic plaque (plaque length 2.25 cm; maximal thickness 0.32 cm). **(C)** B-mode ultrasonography of the RICA after CEA (“ACID pós endarterectomia”), demonstrating a patent arterial lumen without significant residual stenosis. Source: authors' personal archive.

**Figure 2:**
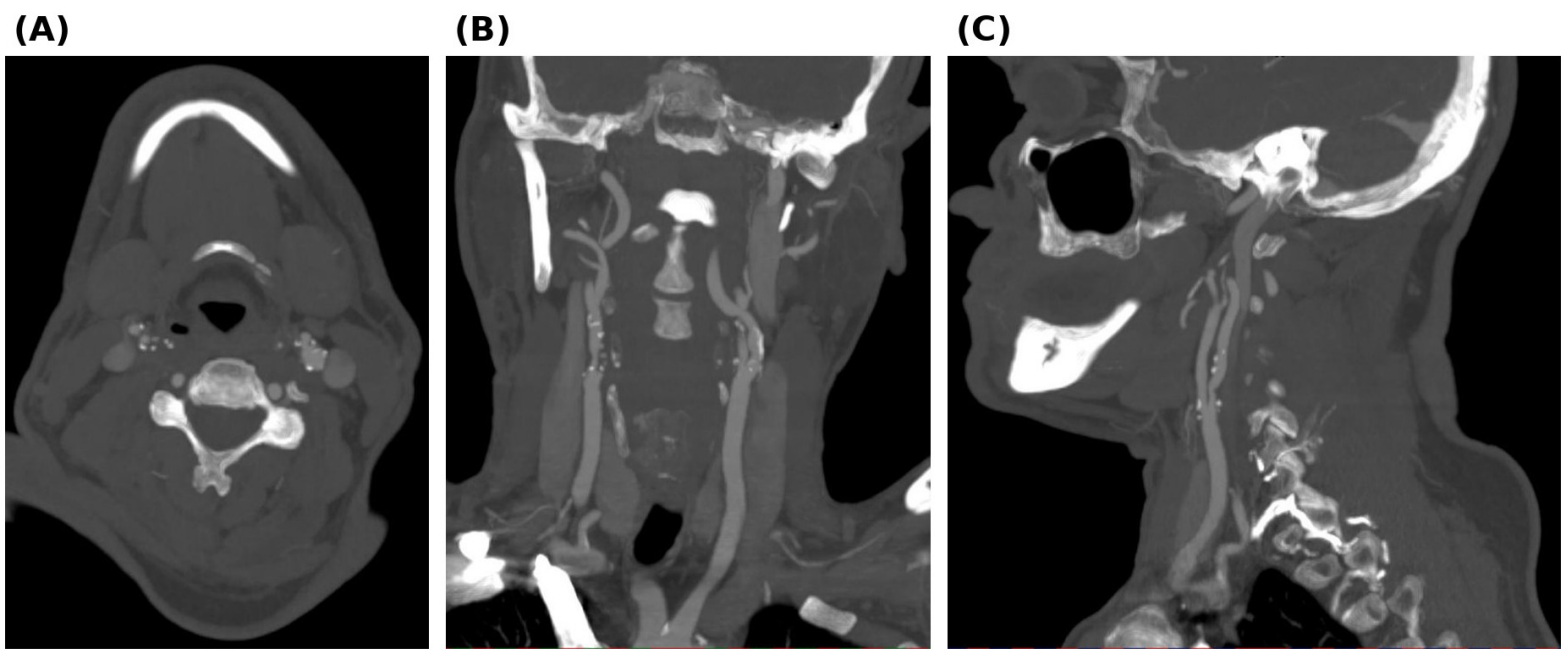
Arterial-phase computed tomography angiography (CTA) of the same patient as in Figure 1, demonstrating 70%−99% stenosis (NASCET) of the right internal carotid artery (RICA). **(A)** Axial reconstruction at the level of the cervical spine. **(B)** Coronal maximum-intensity projection (MIP) of the cervical carotid arteries. **(C)** Sagittal MIP of the right cervical carotid system. Multiplanar CTA reconstruction provides accurate quantification of stenosis severity, plaque morphology, calcification, and lesion length, parameters that are critical for procedural planning. Source: authors' personal archive.

**Figure 3:**
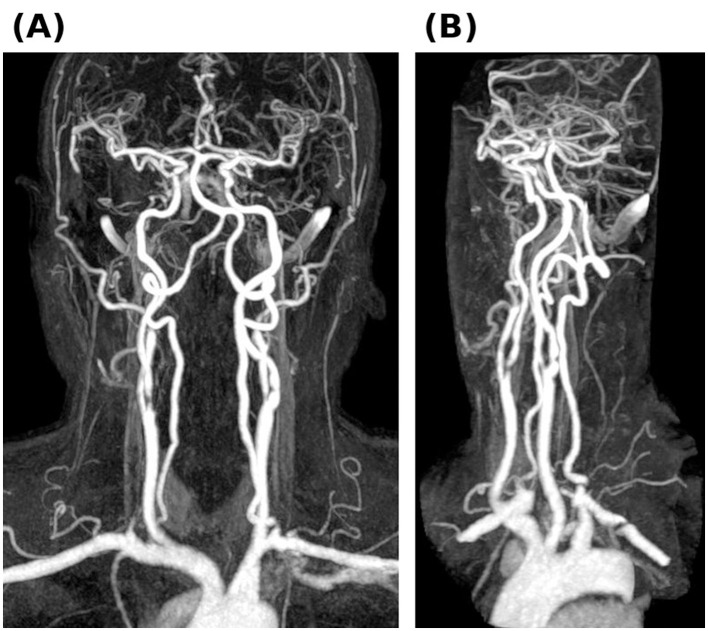
Contrast-enhanced magnetic resonance angiography (CE-MRA, T1-weighted sequences) of the same patient as in Figure 1, demonstrating 70%−99% stenosis (NASCET) of the right internal carotid artery (RICA). **(A)** Coronal maximum-intensity projection (MIP) of the cervical and intracranial arteries. **(B)** Oblique sagittal MIP of the right cervical carotid system. CE-MRA enables simultaneous assessment of the supra-aortic vessels and the intracranial circulation without ionizing radiation, and supports the identification of plaque vulnerability features when combined with high-resolution vessel-wall imaging. Source: authors' personal archive.

**Figure 4:**
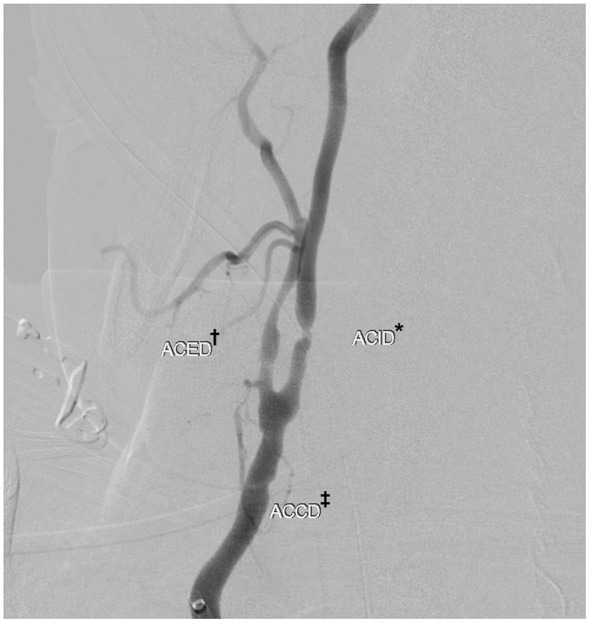
Digital subtraction angiography (DSA) of the same patient as in Figure 1, demonstrating 70%−99% stenosis (NASCET) at the proximal segment of the right internal carotid artery (RICA, *). The right external carotid artery (RECA, †) and right common carotid artery (RCCA, ‡) are also identified. DSA was the imaging modality used to define the NASCET and ECST criteria for grading carotid stenosis and continues to be considered the reference standard against which non-invasive techniques are validated. The original Portuguese labels (ACID, ACED, and ACCD) correspond to the Portuguese acronyms for these arteries (right internal, external, and common carotid arteries, respectively). Source: authors' personal archive.

A representative clinical case illustrating the complementary application of these imaging modalities in the diagnostic assessment of high-grade internal carotid artery stenosis is provided in Figures 1–4, encompassing carotid duplex ultrasonography with spectral Doppler analysis (Figure 1), arterial-phase computed tomography angiography (Figure 2), contrast-enhanced magnetic resonance angiography (Figure 3), and digital subtraction angiography (Figure 4).

### 1.7 Historical evolution of treatment

Carotid endarterectomy was first performed in 1953 and became one of the most extensively evaluated surgical procedures in medicine. The seminal clinical trials conducted in the 1980s and 1990s, including the NASCET (*North American Symptomatic Carotid Endarterectomy Trial*) and the ECST (*European Carotid Surgery Trial*), established the benefit of endarterectomy in the prevention of recurrent stroke in patients with 70%−99% symptomatic carotid stenosis and, to a lesser extent, in subgroups of patients with 50%−69% symptomatic stenosis (North American Symptomatic Carotid Endarterectomy Trial Collaborators, 1991; Warlow, 1991).

For asymptomatic patients, the ACAS (Asymptomatic Carotid Atherosclerosis Study) and ACST (Asymptomatic Carotid Surgery Trial) demonstrated that endarterectomy reduced stroke risk compared with the medical therapy available at that time. In ACAS, endarterectomy reduced the 5-year risk of ipsilateral stroke and any periprocedural stroke or death from 11.0% to 5.1%. ACST-1 confirmed an absolute benefit of 4.6% at 10 years (Walker, 1995; Halliday et al., 2010).

Carotid artery stenting was introduced four decades after endarterectomy and was subsequently evaluated in comparative clinical trials. The major trials comparing the two revascularization techniques in symptomatic patients, including SPACE, EVA-3S, ICSS, and CREST, demonstrated in an individual patient data meta-analysis an absolute increased periprocedural risk of 3.2% of stroke or death with first-generation carotid *stents* compared with endarterectomy. This excess risk appeared to be modified by age, with endarterectomy being safer than *stent* in patients older than 70 years.

The advances in the medical treatment of atherosclerosis, particularly the more widespread use of statins, substantially altered the baseline risk of major vascular events in patients with carotid stenosis, including: ischemic stroke, acute myocardial infarction, cardiovascular death, or the emergence of symptoms necessitating urgent carotid or coronary revascularization. The *Heart Protection Study* demonstrated that allocation to simvastatin 40 mg halved the rate of endarterectomies (0.4% vs. 0.8%; *p* = 0.0003) (Heart Protection Study Collaborative Group, 2002). A clinical trial published in 2020 demonstrated that, in patients with documented cerebrovascular or coronary atherosclerosis, optimizing medical therapy with statins and/or ezetimibe to achieve a serum *Low-Density Lipoprotein Cholesterol* (LDL-c) target below 70 mg/dL significantly reduced the risk of subsequent major vascular events when compared with the 90–110 mg/dL target [*Hazard Ratio* (HR) of 0.78; *p* = 0.04] (Amarenco et al., 2020).

This LDL-c target has been progressively revised downward by international guidelines for patients at very high cardiovascular risk, in alignment with the accumulating evidence that lower LDL-c values translate into greater absolute risk reduction for atherosclerotic cardiovascular events. The ECST-2 trial protocol incorporated a target LDL-c < 77 mg/dL (≈2.0 mmol/L) and systolic blood pressure < 135/85 mmHg (< 145/85 mmHg for patients above 80 years), reflecting the contemporary understanding that optimal medical therapy must achieve substantially tighter risk-factor control than that available during the original NASCET, ECST and ACAS recruitment phases (Amarenco et al., 2020; Donners et al., 2025). The 2025 ESC focused update on dyslipidemia management, the 2025 American Association of Clinical Endocrinology guideline, and the 2026 ACC/AHA dyslipidemia guideline have converged on an LDL-c target < 55 mg/dL (1.4 mmol/L) for patients at very high cardiovascular risk—a classification that explicitly includes individuals with documented carotid stenosis ≥ 50% (Mach et al., 2025; Patel et al., 2025; Blumenthal et al., 2026a). The 2025 Brazilian Guideline adopts an even more stringent target of < 50 mg/dL for this population (Rached et al., 2025). These contemporary thresholds substantially exceed the LDL targets employed in the landmark trials of the 1990s and 2000s, in which statin therapy was inconsistently prescribed and LDL goals, when defined, typically ranged from 100 to 130 mg/dL. The progressive intensification of medical therapy constitutes the principal explanation for the substantial decline in stroke recurrence rates observed in medically managed cohorts over the past two decades (from approximately 4% per year in the early 2000s to approximately 1.5% per year in contemporary populations) and lies at the heart of the current debate regarding the residual role of revascularization in carotid atherosclerotic disease (Rodrigues et al., 2025; Hackam, 2021).

### 1.8 Rationale

The development of an extensive narrative review on the treatment of carotid stenosis is justified by multiple clinical, economic, social, and scientific rationales that converge on the need for systematization and critical analysis of contemporary knowledge in this field.

From a clinical standpoint, carotid stenosis represents one of the leading treatable causes of ischemic stroke, a condition that remains among the leading causes of death and disability worldwide. The CREST-2 data, published in 2025, yielded paradigm-shifting results that challenge concepts established for decades: carotid *stent* combined with OMT demonstrated statistically significant superiority over medical therapy alone, whereas carotid endarterectomy, a procedure considered the gold standard for over three decades, did not achieve statistical significance in this same comparison (Brott et al., 2026). These findings demand critical reassessment of current therapeutic strategies and guideline recommendations.

The economic relevance of this review relates to the substantial costs associated with both cerebrovascular events and revascularization procedures. A disabling stroke entails direct costs for hospitalization, rehabilitation, and long-term care, in addition to indirect costs related to productivity losses. The adequate identification of which patients benefit from preventive interventions and which treatment modality offers the best cost-effectiveness has significant implications for healthcare systems with limited resources.

The social dimension of this issue is evidenced by the impact of stroke on the quality of life of patients and their families and caregivers. Stroke constitutes the leading cause of acquired disability in adults, and the prevention of events through adequate treatment of carotid stenosis represents an opportunity for substantial reduction of this social burden.

From a scientific perspective, the simultaneous publication of two highly relevant clinical trials in 2025, CREST-2 and ECST-2, with apparently divergent conclusions, creates the need for an integrated and contextualized analysis of these data (Brott et al., 2026; Donners et al., 2025). ECST-2 did not demonstrate a benefit of revascularization added to optimal medical therapy in its 2-year interim analysis, whereas CREST-2 demonstrated a benefit of carotid *stent* at 4-year follow-up (Brott et al., 2026; Donners et al., 2025). The reconciliation of these findings requires careful methodological analysis of the differences between the studies, including selection criteria, endpoint definitions, follow-up duration, and characteristics of the medical treatment employed.

Additionally, the update of the European Society for Vascular Surgery guidelines in 2023 and the clinical consensus statement on stroke risk management in carotid atherosclerotic disease endorsed by the European Society of Cardiology reinforce the need for a systematic review integrating these new recommendations with the most recent data from randomized clinical trials (RCT).

### 1.9 Objectives

The objective of this study is to provide an extensive and rigorous narrative review on the treatment of carotid stenosis, encompassing the common and internal carotid arteries.

Specifically, this review aims to:

(a) Critically analyze the available scientific evidence on the three main therapeutic modalities for carotid stenosis: optimal medical therapy, carotid endarterectomy, and carotid artery *stent*;
(b) Synthesize the results of the major RCTs, with particular emphasis on the paradigm-shifting studies published in 2025, including CREST-2 and ECST-2;
(c) Compare the favorable and unfavorable outcomes of each therapeutic approach, rigorously grounded in the available literature data;
(d) Discuss the practical clinical implications of the most recent findings, identifying potential paradigm shifts in the approach to asymptomatic and symptomatic carotid stenosis;
(e) Identify gaps in current knowledge that require investigation in future research.

## 2 Methods

### 2.1 Study design

This study constitutes a narrative review of the scientific literature on the treatment of atherosclerotic carotid stenosis. The narrative review was selected as the method because it allows for a broad and integrative analysis of a complex topic, incorporating different types of evidence and enabling contextualized critical discussion of the findings.

### 2.2 Search strategy

The literature search was conducted in the following electronic databases: PubMed/MEDLINE, LILACS-VHL (Latin American and Caribbean Health Sciences Literature/Virtual Health Library), Google Scholar and SciELO (Scientific Electronic Library Online).

The descriptors used were selected from the DeCS (Health Sciences Descriptors) and MeSH (Medical Subject Headings) systems, corresponding to the keywords of this study: “Carotid Stenosis” (Carotid Stenosis), “Carotid Endarterectomy” (Carotid Endarterectomy), “Stents”, “Stroke” and “Atherosclerosis.” Combinations of these descriptors were employed using Boolean operators (AND, OR) to optimize the retrieval of relevant articles.

### 2.3 Inclusion and exclusion criteria

Studies published between 2020 and 2025 were included, aiming to address the most recent evidence on the topic. No restrictions were applied regarding language or regional origin for the article search, ensuring maximum comprehensiveness of the review. RCTs, meta-analyses, systematic reviews, observational cohort studies, and clinical registries were selected, as well as medical society guidelines and expert consensus statements relevant to the topic.

Studies of greatest scientific impact and clinical relevance were prioritized, with particular emphasis on the most recent publications that provided important information to be considered in clinical practice.

### 2.4 Selection process

Article selection was performed in two stages. In the first stage, titles and abstracts were evaluated to identify potentially eligible publications. In the second stage, pre-selected articles were read in full to assess their relevance and methodological quality. The search was supplemented by reviewing the reference lists of included articles to identify additional relevant publications.

### 2.5 Ethical considerations

As this is a literature review that exclusively uses previously published secondary data, with no involvement of primary patient data or any intervention in human subjects, this study is exempt from review by a Research Ethics Committee, in accordance with Resolution No. 510/2016 of the Brazilian National Health Council.

## 3 Subsections relevant for the subject

### 3.1 Historical clinical trials in symptomatic carotid stenosis

The contemporary management of symptomatic carotid stenosis is based on the results of trials conducted over three decades ago. The NASCET and ECST established the benefit of carotid endarterectomy in the prevention of recurrent stroke in patients with high-grade symptomatic stenosis—defined as ≥70% in NASCET and ≥80% in ECST. In these trials, endarterectomy carried a periprocedural risk of stroke and death of approximately 7% and did not prevent stroke recurrence in all patients, suggesting that surgery should be avoided in patients at low risk of stroke under medical therapy alone (North American Symptomatic Carotid Endarterectomy Trial Collaborators, 1991; Warlow, 1991).

The pooled analysis of individual patient data from 6,092 patients in the NASCET, ECST, and *Veterans Affairs* trials demonstrated that multiple factors beyond the degree of stenosis, including age, sex, time since the index event, and plaque morphology, influence the risk of future stroke and, consequently, the absolute benefit of intervention. Patients with a predicted 5-year risk of ipsilateral stroke exceeding 20% derived substantial benefit from endarterectomy in NASCET, whereas those with a lower predicted risk did not benefit (Donners et al., 2025).

### 3.2 Historical clinical trials in asymptomatic carotid stenosis

The endarterectomy trials for asymptomatic carotid stenosis were also conducted over 30 years ago. ACAS demonstrated a reduction in the 5-year risk of ipsilateral stroke and any periprocedural stroke or death from 11.0% to 5.1% with endarterectomy. ACST-1 subsequently confirmed an absolute benefit of 4.6% at 10 years. Follow-up data of 10–15 years from ACST-1 in patients receiving triple therapy (antiplatelet, statin, and antihypertensive) before any stroke showed no loss of the initial benefit, and stroke risk curves continued to diverge, consistent with a durable benefit of revascularization over medical therapy (Walker, 1995; Halliday et al., 2010).

However, it is essential to recognize that the medical treatment used in these historical trials was substantially different from contemporary standards. The evolution of pharmacological therapy, particularly the more widespread and intensive use of statins, antiplatelet agents, and antihypertensives, has significantly altered the baseline risk of events in patients with carotid stenosis.

### 3.3 The CREST-2 trial

The CREST-2 (*Carotid Revascularization and Medical Management for Asymptomatic Carotid Stenosis Trials*), published in the *New England Journal of Medicine* in 2025, comprised two parallel, observer-blinded trials that randomized patients with high-grade asymptomatic carotid stenosis (≥70%) across 155 centers in five countries (Brott et al., 2026). The main recruitment phase and the outcome assessment have been completed, with landmark publications detailing the benefit of optimized medical therapy. Some extension studies remain under follow-up.

The *stent* trial compared optimal medical therapy alone vs. *stent* combined with OMT. The endarterectomy trial compared optimal medical therapy alone vs. endarterectomy combined with OMT. The OMT targets included: blood pressure control [initially targeting systolic blood pressure (SBP) below 140 mmHg, later reduced to < 130 mmHg following updates in the 2018 American Hypertension Guideline], LDL-c < 70 mg/dL, glycemic control, smoking cessation, weight management, and avoidance of a sedentary lifestyle. The composite primary endpoint included any stroke or death, assessed from the time of randomization through 44 days, or ipsilateral ischemic stroke, assessed during the remaining follow-up through 4 years (Brott et al., 2026).

A total of 1,245 patients were randomized in the *stent* trial and 1,240 in the endarterectomy trial. Eligibility criteria included age 35 years or older, no history of stroke, TIA, or amaurosis fugax in the carotid territory within the 180 days preceding randomization, and stenosis of at least 70% documented by Doppler ultrasound (peak systolic velocity ≥ 230 cm/s) with additional confirmation by other velocity criteria or by CTA/MRA/conventional angiography (Brott et al., 2026).

#### 3.3.1 Results of the stenting trial (CREST-2)

In the *stent* trial of CREST-2, the incidence of primary endpoint events at 4 years was 6.0% (95% CI, 3.8–8.3) in the medical therapy alone group and 2.8% (95% CI, 1.5–4.3) in the *stent* group. The absolute risk difference was 3.2 percentage points (95% CI, 0.2–6.2; *p* = 0.02), with a relative risk of 2.15 (95% CI, 1.12–4.44) for the medical therapy group compared with the *stent* group. The number needed to treat was 31 to prevent one primary endpoint event over 4 years (Brott et al., 2026).

During the periprocedural period (day 0 through 44), no stroke or death occurred in the OMT alone group, whereas seven strokes and one death occurred in the *stenting group*. After 44 days, 28 ipsilateral ischemic strokes occurred among 600 patients followed for 1,759 person-years in the medical therapy group, corresponding to an annual rate of 1.6% (95% CI, 1.1–2.3). In the *stent* group, 11 strokes occurred among 582 patients followed for 1,851 person-years, corresponding to an annual rate of 0.6% (95% CI, 0.3–1.1), with a relative risk of 2.67 (95% CI, 1.33–5.61) for medical therapy compared with *stent* (Brott et al., 2026).

#### 3.3.2 Results of the endarterectomy trial (CREST-2)

In the endarterectomy trial of CREST-2, the incidence of primary endpoint events at 4 years was 5.3% (95% CI, 3.3–7.4) in the medical therapy alone group and 3.7% (95% CI, 2.1–5.5) in the endarterectomy group. The absolute risk difference was 1.6 percentage points (95% CI, −1.1 to 4.3; *p* = 0.24), and the relative risk was 1.43 (95% CI, 0.78–2.72) for the medical therapy group compared with endarterectomy. These results did not achieve statistical significance (Brott et al., 2026).

During the periprocedural period, three strokes occurred in the medical therapy group (0.5%; 95% CI, 0.1–1.4) and nine strokes in the endarterectomy group (1.5%; 95% CI, 0.7–2.8). After 44 days, 23 ipsilateral ischemic strokes occurred among 600 patients followed for 1,761 person-years in the medical therapy group, corresponding to an annual rate of 1.3% (95% CI, 0.9–2.0). In the endarterectomy group, 10 strokes occurred among 596 patients followed for 1,823 person-years, corresponding to an annual rate of 0.5% (95% CI, 0.3–1.0), with a relative risk of 2.38 (95% CI, 1.13–5.00) (Brott et al., 2026).

### 3.4 The ECST-2 trial

The ECST-2 (*Second European Carotid Surgery Trial*), still ongoing, is a randomized, multicenter clinical trial conducted at 30 centers with expertise in stroke and carotid revascularization in Europe and Canada. The study was terminated before reaching the original target of 2,000 patients due to recruitment and funding difficulties, but the researchers published provisional data (Nederkoorn and De Borst, 2025). Eligible individuals were those aged 18 years or older, with asymptomatic or symptomatic carotid stenosis with stenosis of 50% or more of the vascular lumen, and with a predicted 5-year risk of ipsilateral stroke below 20% (estimated by the *Carotid Artery Risk*—CAR score) (Donners et al., 2025).

The patients were randomized to optimal medical therapy (OMT) alone or OMT plus revascularization (1:1). The primary endpoint for the 2-year interim analysis was a hierarchical composite that included: (1) periprocedural death, fatal stroke, or fatal myocardial infarction; (2) non-fatal stroke; (3) non-fatal myocardial infarction; (4) new silent cerebral infarction on imaging (Donners et al., 2025).

The ECST-2 defined OMT as: a low-cholesterol diet, use of lipid-lowering medications targeting < 77 mg/dL; blood pressure control with a target of < 135/85 mmHg or < 145/85 mmHg in patients over 80 years of age; smoking cessation; as well as glycemic and body weight control.

Between March 2012 and October 2019, 429 patients were randomized to OMT alone (*n* = 215) or OMT plus revascularization (*n* = 214). The median age was 72 years and 69% were male. No benefit was recorded in favor of either treatment group regarding the hierarchical primary endpoint at 2 years, with 5,228 (11.4%) wins for the OMT alone group, 5,173 (11.3%) wins for the OMT plus revascularization group, and 35,395 (77.3%) ties between groups (*win ratio* 1.01; 95% CI 0.60–1.70; *p* = 0.97 vs. 0.97) (Donners et al., 2025).

In 2025, a 2-year interim analysis of the study was published in The Lancet Neurology. For OMT alone vs. OMT plus revascularization, respectively: 4 vs. 3 patients experienced periprocedural death, fatal stroke, or fatal myocardial infarction; 11 vs. 16 had non-fatal stroke; 7 vs. 5 had non-fatal myocardial infarction; and 12 vs. 7 presented new silent cerebral infarction on imaging (Brott et al., 2026). One periprocedural death occurred in the revascularization group, attributed to decompensated aortic stenosis 1 week after carotid endarterectomy (Donners et al., 2025).

The chronological and comparative synthesis of the main randomized clinical trials on the treatment of carotid stenosis, from the foundational studies of the 1990s to the paradigm-shifting trials of 2025–2026, is presented in Table 2. This consolidated overview provides the integrated visual reference upon which the critical interpretation developed in the subsequent sections is built.

**Table 2:** Chronological and comparative synthesis of the main randomized clinical trials on the treatment of carotid stenosis, highlighting methodological evolution and the results of comparisons between endarterectomy, stenting, and optimal medical therapy.

Trial	Year of publication	Population	Comparison	Primary endpoint (summary)	Main result
NASCET	1991	Symptomatic 70%−99%	CEA^*^+ ASA^**†**^ vs. ASA alone	Ipsilateral IS^**‡**^ at 2 years	ARR^**§**^ 17%; NNT^**¶**^ 6 (NASCET 70%−99%)
ECST	1998	Symptomatic ≥80% (ECST)	CEA + ASA vs. ASA alone	IS/death at 3 years	Benefit for severe stenoses; no benefit if < 50%
ACAS	1995	Asymptomatic ≥60%	CEA + OMT^**^ vs. OMT	Ipsilateral IS at 5 years	Reduction from 11.0% to 5.1%
ACST-1	2004	Asymptomatic ≥60%	CEA + OMT vs. OMT	IS/death at 5–10 years	Absolute benefit of 4.6% at 10 years
SPACE	2006	Symptomatic ≥50% (NASCET)	tfCAS vs. CEA	Ipsilateral IS/death at 30 days	Non-inferiority of tfCAS not demonstrated (6.84% vs. 6.34%)
EVA-3S	2006	Symptomatic ≥60%	tfCAS^**††**^ vs. CEA	IS/death at 30 days	Halted prematurely: tfCAS inferior (9.6% vs. 3.9%)
ICSS	2010/2015	Symptomatic ≥50%	tfCAS vs. CEA	Disabling IS/death at 5 years	Long-term equivalence; excess minor events with tfCAS
CREST-1	2010	Symptomatic/ asymptomatic ≥50%−60%	tfCAS vs. CEA	Composite IS/MI^**‡‡**^/death + ipsilateral IS at 4 years	No overall difference; tfCAS superior <70 years, CEA superior >70 years (Voeks et al., 2011)
ACST-2	2021	Asymptomatic ≥ 60%	tfCAS vs. CEA	Death/MI/IS at 30 days and 5 years	No significant overall difference
CREST-2	2026	Asymptomatic ≥ 70%	tfCAS + OMT vs. OMT; CEA + OMT vs. OMT	Composite IS/death + ipsilateral IS at 4 years	tfCAS + OMT superior to OMT (*p* = 0.02); CEA + OMT not significant (*p* = 0.24)
ECST-2	2025 (2-year interim)	Asymptomatic/ symptomatic low-risk ≥50%	Revascularization + OMT vs. OMT	Hierarchical composite (death, IS, MI, silent infarct)	No difference between groups (win ratio 1.01)

Sources: Synthesis prepared by the authors from Brott et al. (2026); Donners et al. (2025); Bierens et al. (2025); Müller et al. (2020); Halliday et al. (2021); Voeks et al. (2011); and historical references (; Warlow, 1991; Walker, 1995; Halliday et al., 2010).

^*^CEA, carotid endarterectomy; ^†^ASA, acetylsalicylic acid; ^‡^IS, ischemic stroke; ^§^ARR, absolute risk reduction; ^¶^NNT, number needed to treat; ^**^OMT, optimal medical therapy; ^†^†tfCAS, transfemoral carotid stenting; ^‡^‡MI, myocardial infarction; LDL-c, LDL cholesterol.

### 3.5 Contemporary optimal medical therapy

Contemporary OMT for carotid stenosis comprises multifactorial pharmacological intervention and lifestyle modification. Fundamental components include antiplatelet therapy, high-intensity statins targeting LDL-c below 70 mg/dL, stringent blood pressure control, and smoking cessation. Observational studies suggest that the risk of stroke related to carotid stenosis may have decreased to approximately 1% per year in general populations under current medical therapy (Constantinou et al., 2013; Dharmakidari et al., 2017; Chang et al., 2022).

An analysis of 864 individuals with asymptomatic carotid stenosis ≥50% evaluated with Doppler ultrasonography on at least three occasions demonstrated that optimal medical therapy reduced the risk of disease progression and ischemic events during long-term follow-up (Shah et al., 2017). However, residual risk persists even in patients adherent to pharmacological therapy, and strokes secondary to carotid atherosclerotic disease continue to occur in adequately treated patients. This may be attributable to the fact that the more intensive blood pressure targets [SBP < 120 or between 120 and 129 mmHg and diastolic blood pressure (DBP) < 80 mmHg (Jones et al., 2025; Brandão et al., 2025; Blumenthal et al., 2026b; van der Born et al., 2026)] and LDL-c targets [<55 mg/dL or < 50 mg/dL (Mach et al., 2025; Patel et al., 2025; Blumenthal et al., 2026a; Rached et al., 2025)] defined by the most recently published guidelines have not yet been incorporated into studies conducted specifically with patients harboring carotid stenosis.

Recent evidence has progressively challenged the historical paradigm that revascularization should be the default treatment for symptomatic carotid stenosis. A systematic review and meta-analysis published in 2025 (Rodrigues et al., 2025), which included five studies (*n* = 1,066 symptomatic patients with carotid stenosis ranging from 50 to 99%), reported pooled long-term stroke risks of 6.96% (95% CI, 4.76%−9.15%) with OMT alone, in contrast with 4.51% (95% CI, 2.67%−6.35%) with revascularization. Corresponding pooled risks of death were 3.14% (95% CI, 1.64%−4.64%) and 2.65% (95% CI, 1.23%−4.08%), respectively, and pooled combined stroke-or-death risks were 8.91% (95% CI, 6.49%−11.33%) vs. 6.56% (95% CI, 4.37%−8.76%). Although a numerical trend favored revascularization, the differences were not statistically significant in most pooled analyses, and the authors concluded that contemporary OMT may constitute a viable alternative to revascularization in selected low-risk symptomatic patients (Rodrigues et al., 2025). A noteworthy exception was the subgroup of carotid near-occlusion (95%−99% stenosis), in which revascularization conferred a significant absolute benefit, with pooled stroke risk of 13.46% (95% CI, 7.46%−19.47%) under OMT alone vs. 2.80% (95% CI, 0.87%−4.72%) with endarterectomy (Rodrigues et al., 2025).

This conclusion gains further weight from the 2-year interim analysis of ECST-2, which similarly failed to demonstrate benefit of revascularization over OMT alone in symptomatic patients with predicted low-to-intermediate stroke risk by the Carotid Artery Risk (CAR) score, and from the contemporary systematic review of Bierens et al. (2025), which analyzed 16 international guidelines on carotid revascularization in symptomatic patients from 12 regions. Bierens et al. (2025) identified substantial discrepancies among recommendations for moderate (50%−69%) symptomatic stenosis: although all 16 guidelines endorse revascularization for at least a subgroup of these patients, only 63% (10 of 16) provide strong recommendations, and 25% (4 of 16) explicitly restrict the indication to male patients, citing limited or absent benefit in female patients. For severe symptomatic stenosis (70%−99%), guideline consensus remains uniformly strong. Notably, all current guidelines derive their core recommendations from three randomized trials conducted three decades ago—NASCET, ECST, and the Veterans Affairs Cooperative Study Program 309—an evidence base that does not incorporate the substantial advances in optimal medical therapy nor the technological evolution of revascularization procedures (Bierens et al., 2025).

In this evolving context, an emerging conceptual framework proposes that carotid endarterectomy, traditionally regarded as the unequivocal gold standard for symptomatic stenosis, should be increasingly reserved for high-risk patient subsets identified through individualized risk stratification—combining the degree of stenosis with plaque vulnerability biomarkers (intraplaque hemorrhage on MRI, ulcerated or echolucent plaque on ultrasound, ipsilateral silent infarcts on cerebral imaging), transcranial Doppler microembolic signal detection, and validated clinical risk scores. For low-risk symptomatic patients and the majority of asymptomatic patients, contemporary intensive OMT may constitute the most appropriate first-line strategy, with revascularization deferred until either symptom recurrence under medical therapy or the identification of plaque-based high-risk features (Paraskevas and AbuRahma, 2024; Bierens et al., 2025; Rodrigues et al., 2025; Hackam, 2021).

### 3.6 Transcarotid artery revascularization (TCAR)

#### 3.6.1 Concept, procedural rationale and regulatory trajectory

Transcarotid artery revascularization (TCAR) is a hybrid carotid revascularization technique that combines a brief cervical exposure of the common carotid artery (CCA) with endovascular stent delivery performed under continuous dynamic cerebral flow reversal. Neuroprotection is established *before* any contact with the atherosclerotic lesion through the ENROUTE™ Transcarotid Neuroprotection System (Silk Road Medical, Sunnyvale, CA, USA), which creates an extracorporeal arteriovenous circuit between the CCA and the common femoral vein, reversing the internal carotid artery (ICA) flow direction and diverting any embolic debris away from the cerebral circulation (Dodwad et al., 2026; Kwolek et al., 2015). This design directly addresses the two major embolic sources historically responsible for the excess periprocedural stroke risk of transfemoral CAS (tfCAS)—manipulation of the aortic arch and unprotected crossing of the lesion for distal filter deployment (Dodwad et al., 2026; Naazie et al., 2020). Initially cleared by the U.S. Food and Drug Administration in 2015 for patients deemed at high anatomical or physiological risk for carotid endarterectomy (CEA), TCAR had its indication broadened in May 2022 to include standard-risk individuals, and the Centers for Medicare and Medicaid Services (CMS) extended reimbursement to all forms of CAS, including TCAR, in October 2023 (Dodwad et al., 2026; Jim et al., 2026).

The contemporary diffusion of TCAR across vascular practice has been quantitatively characterized by Columbo et al. (2023), who analyzed the patterns of adoption since FDA approval and documented an exponential increase in procedural volume, accompanied by progressive expansion of the operator base and broadening of the participating centers. This pattern of rapid uptake (atypical for a procedure approved without supporting evidence from a randomized clinical trial) reflects both the favorable safety signal accumulated through mandatory enrolment in the Vascular Quality Initiative (VQI) registry and the technical accessibility of the procedure for surgeons already familiar with carotid surgery, in contrast to the comparatively steeper learning curve of transfemoral carotid stenting. The authors highlight, however, that adoption has not been geographically uniform, with concentration in high-volume centers and persistent variability in operator experience, factors that should temper the extrapolation of trial-level outcomes to community practice (Columbo et al., 2023).

#### 3.6.2 Pivotal prospective evidence: the ROADSTER program

The clinical foundation of TCAR rests on three sequential, industry-monitored prospective trials. ROADSTER (2015) enrolled 141 high-risk patients across 18 sites and reported a technical success rate of 99.3% with only two minor strokes (1.4%), two deaths and one myocardial infarction (MI) within 30 days (Kwolek et al., 2015). ROADSTER 2 (2020), a post-approval study designed by FDA directive with over 70% of operators being TCAR-naïve, recruited 632 high-risk patients and demonstrated 99.7% technical success, 0.6% periprocedural stroke, 0.2% mortality, 0.9% MI and a composite 30-day stroke/death/MI (S/D/MI) rate of 1.7% (Kashyap et al., 2020). Finally, ROADSTER 3 (2024) [the first independently adjudicated prospective trial of TCAR in standard-risk patients (*n* = 344 intention-to-treat; *n* = 320 per-protocol)] reported a composite 30-day S/D/MI rate of 0.6% (per-protocol) and 0.9% (intention-to-treat), no deaths and no MIs, and a S/D/MI rate of 0% in symptomatic patients, which represents to date the lowest periprocedural stroke rate documented in any independently adjudicated prospective carotid stenting trial (Müller et al., 2020). Cranial nerve injury (CNI), a recognized morbidity of CEA, occurred in 0.6% of patients, well-below the prespecified threshold of 2.7% (*p* = 0.0077) (Jim et al., 2026).

#### 3.6.3 Real-world comparative effectiveness

Large registry analyses and propensity-matched cohort studies have provided externally valid corroboration of these prospective findings. In a single-institution series of 1,000 consecutive TCAR procedures performed by 23 surgeons between 2017 and 2025, Dodwad et al. (2026) reported 30-day rates of 1.9% for stroke, 1.0% for death and 0.2% for MI (composite 2.9%), with a median length of stay of 1 day; prior stroke [adjusted odds ratio (aOR) 2.4; *p* < 0.001] and chronic obstructive pulmonary disease (aOR 1.7; *p* = 0.011) were identified as independent predictors of perioperative S/D, whereas perioperative clopidogrel (aOR 0.45; *p* = 0.014) and intraoperative protamine (aOR 0.47; *p* = 0.011) were protective (Dodwad et al., 2026). An updated 7-year analysis of the Society for Vascular Surgery Vascular Quality Initiative (SVS-VQI) database (*n* = 50,068 TCAR; 25,361 tfCAS; 122,737 CEA) confirmed that TCAR is substantially safer than tfCAS regarding stroke or death (1.6% vs. 2.9%; aOR 0.54; 95% CI, 0.49–0.61) and only marginally inferior to CEA (1.6% vs. 1.3%; aOR 0.83; 95% CI, 0.76–0.91), while presenting a markedly lower CNI rate than CEA (0.3% vs. 2.3%) (Straus et al., 2024).

A target-trial-emulation analysis of the Nationwide Readmissions Database (*n* = 113,994 CEA; 12,613 TCAR) demonstrated equivalent 90-day composite morbidity between TCAR and CEA (4.1% vs. 4.1%), with a reduced hazard of MI specifically among high-risk patients [hazard ratio (HR) 0.76; 95% CI, 0.58–0.98] (Sakowitz et al., 2025). Three-year follow-up data from Medicare beneficiaries (*n* = 19,141 asymptomatic and 12,343 symptomatic) showed that the cumulative stroke risk after TCAR remains comparable to CEA in both clinical presentations, with an adjusted HR of 1.10 (95% CI, 0.82–1.49) for asymptomatic patients (Columbo et al., 2025). A meta-analysis encompassing 45 studies and 14,588 patients further consolidated these data, reporting a 99% technical success rate, 1.3% periprocedural stroke, 0.5% mortality and 1.5% in-stent restenosis (Sagris et al., 2021), whereas the propensity-matched analysis by Malas et al. (2022) showed no significant differences in 1-year ipsilateral stroke or death between TCAR and CEA (6.49% vs. 5.68%; HR 1.14; 95% CI, 0.95–1.37).

The chronological synthesis of the three pivotal prospective studies of the ROADSTER program, which collectively define the contemporary evidence base for transcarotid artery revascularization, is presented in Table 3. Because these are single-arm prospective studies, they are presented separately from Table 2 to preserve methodological transparency and to avoid inappropriate direct comparisons with the randomized trials listed therein.

**Table 3:** Chronological synthesis of the pivotal prospective studies of the ROADSTER program on transcarotid artery revascularization (TCAR), with emphasis on 30-day periprocedural outcomes.

Trial	Year	Population	Design	Primary endpoint (summary)	Main result
**ROADSTER** (Kwolek et al., 2015)	2015	High surgical risk for CEA (*n* = 141; 18 sites)	Prospective, multicenter, single-arm	Periprocedural S/D/MI^**†**^ (≤30 days)	Technical success 99.3%; stroke 1.4%; death 1.4%; MI^**‡**^ 0.7%
**ROADSTER 2** (Kashyap et al., 2020)	2020	High surgical risk for CEA (*n* = 632; >70% TCAR-naïve operators)	FDA^**§**^ post-approval; prospective, multicenter, single-arm	Periprocedural S/D/MI (≤30 days)	Technical success 99.7%; stroke 0.6%; death 0.2%; MI 0.9%; composite 1.7%
**ROADSTER 3** (Jim et al., 2026)	2026	Standard surgical risk for CEA (*n* = 344 ITT^**¶**^; *n* = 320 per-protocol)	Prospective, multicenter, independently adjudicated, single-arm	Periprocedural S/D/MI (≤30 days)	S/D/MI 0.6% (PP); 0.9% (ITT); 0% stroke in symptomatic patients; no deaths; no MIs; CNI^**^ 0.6%

Sources: Synthesis prepared by the authors from Kwolek et al. (2015); Kashyap et al. (2020); and Jim et al. (2026).

ROADSTER 3 represents, to date, the lowest periprocedural stroke rate documented in any independently adjudicated prospective carotid stenting trial (Kwolek et al., 2015). ^*^CEA, carotid endarterectomy; ^†^S/D/MI, composite of stroke/death/myocardial infarction; ^‡^MI, myocardial infarction; ^§^FDA, U.S. Food and Drug Administration; ^¶^TT, intention-to-treat; ^**^CNI, cranial nerve injury; ^*^CEA, carotid endarterectomy.

#### 3.6.4 Evidence in symptomatic patients

A study-level systematic review and meta-analysis by Ghannam et al. (2024), published in Stroke in 2024, specifically addressed the comparative safety and efficacy of TCAR in patients with symptomatic internal carotid artery disease, an evidentiary niche scarcely represented in the pivotal prospective trials. In this analysis, encompassing studies published between January 2000 and February 2023, TCAR demonstrated comparable 30-day rates of stroke, transient ischemic attack, myocardial infarction and mortality when contrasted with CEA in the symptomatic subgroup, with a more favorable safety profile than transfemoral CAS. Importantly, TCAR was associated with a markedly lower rate of cranial nerve injury than CEA, a finding consistent with the broader real-world literature. The authors concluded that, although prospective randomized trials are still needed to consolidate the role of TCAR in symptomatic disease, the available evidence supports the consideration of TCAR as a viable alternative to CEA and CAS in this clinically distinct subgroup, particularly when anatomical or physiological characteristics render CEA technically challenging (Ghannam et al., 2024).

#### 3.6.5 Position of TCAR within the therapeutic algorithm, or as a complement to it

A comprehensive contemporary appraisal of the therapeutic role of TCAR has been offered by Zarrintan and Malas (2023), who integrated the regulatory trajectory, the cumulative procedural evidence and the comparative effectiveness data into a unified conceptual framework. The authors emphasize that TCAR has progressively evolved from a niche option for patients deemed at high anatomical or physiological risk for CEA into a third therapeutic pillar that may legitimately displace transfemoral CAS whenever an endovascular approach is judged appropriate. They underscore that the favorable periprocedural safety profile of TCAR, particularly with respect to stroke and cranial nerve injury, is mechanistically supported by the avoidance of aortic arch manipulation and by the establishment of cerebral neuroprotection before any contact with the atherosclerotic lesion. Nevertheless, in the absence of a head-to-head randomized comparison with CEA, the hierarchical status of CEA as the surgical reference standard remains conceptually intact for the broad symptomatic population, with TCAR positioned as a complementary modality whose precise place in the therapeutic algorithm is still being defined (Zarrintan and Malas, 2023).

## 4 Discussion

### 4.1 Interpretation of CREST-2 results

The results of the *stent* trial of CREST-2 represent a paradigm shift in the understanding of asymptomatic carotid stenosis treatment. For the first time in a large-scale RCT, the addition of a revascularization procedure to optimal medical therapy demonstrated statistically significant superiority over medical therapy alone in patients with high-grade asymptomatic stenosis (Brott et al., 2026).

The benefit observed in the *stent* trial was consistent across prespecified subgroups, including analyses by age, sex, stenosis severity, presence of comorbidities, and prior history of cerebrovascular events. The absolute risk difference of 3.2 percentage points over 4 years translated into a number needed to treat (NNT) of 31, that is: approximately 31 patients would need to undergo carotid *stent* in addition to OMT to prevent one primary endpoint event.

Conversely, the endarterectomy trial of CREST-2 did not demonstrate statistically significant benefit of adding this surgical procedure to optimal medical therapy, with an absolute risk difference of only 1.6 percentage points, failing to achieve statistical significance (*p* = 0.24). This finding challenges decades of clinical practice grounded in the ACAS and ACST-1 trials, which established endarterectomy as the standard of treatment for asymptomatic carotid stenosis (Brott et al., 2026).

The sensitivity analysis (*tipping-point analysis*) of the endarterectomy trial suggested that statistical significance would have been achieved if seven or more events were removed from the endarterectomy group or if six or more events were added to the medical therapy group, indicating that the study was relatively close to demonstrating significant benefit (Brott et al., 2026). This, together with the fact that the control arm of the endarterectomy trial achieved better outcomes than the control arm of the *Stent* trial, underscores the fragility of the statistical power of the study.

During the periprocedural period, a total of seven strokes occurred in patients who underwent *stent* (1.3%), which represents a relatively low incidence, suggesting that the medical institutions involved in the study likely have lower complication rates compared with those documented at most healthcare institutions (Paraskevas and AbuRahma, 2024; Constantinou et al., 2013). Therefore, it is presumed that these findings are not generalizable to most medical centers performing the procedure worldwide (Brott et al., 2026; Brown and Bonati, 2026).

The Supplementary Appendix of the CREST-2 trial (Brott et al., 2026) demonstrate that a significant proportion of patients were unable to adhere to the OMT proposed by the investigators. Approximately 40% did not achieve blood pressure targets throughout the study and, over a 12-month period, 30.7% did not reach the LDL-c target. Furthermore, only 50% successfully achieved the glycemic control benchmark and, within the same 12-month interval, 21.1% had not ceased tobacco use (Brott et al., 2026). These limitations lead us to consider that the actual benefit of OMT may have been underestimated by the study results (Brott et al., 2026; Brown and Bonati, 2026).

When comparing the arms of CREST-2, it is noteworthy that the control group of the *stent* arm had a stroke rate of 6%, while the control group of the endarterectomy arm had a rate of 5.3%. This difference, although small, may also have influenced the statistical significance in the *stent* group and the lack of significance in the endarterectomy group. A future individual patient data meta-analysis may be useful to elucidate the extent to which this variation between control groups may have influenced the final result of each arm, adjusting for the difference between controls.

### 4.2 Critical analysis of ECST-2

The 2-year interim results of ECST-2 did not demonstrate evidence of benefit from revascularization in addition to OMT in patients with asymptomatic or symptomatic carotid stenosis of 50% or more with a predicted 5-year stroke risk below 20%. The analysis using the *win ratio* method, novel in carotid stenosis trials, resulted in a virtual tie between treatment groups (Donners et al., 2025).

However, several methodological considerations are necessary when interpreting these results in comparison with CREST-2. First, ECST-2 included patients with a lower degree of stenosis (≥50%) and, crucially, patients with a low predicted stroke risk by the CAR score, a population in which the benefit of revascularization would naturally be smaller. Second, a follow-up of only 2 years may be insufficient to detect benefits of preventive interventions, considering that the event curves in CREST-2 continued to diverge throughout 4 years of follow-up (Brott et al., 2026; Donners et al., 2025).

The ECST-2 authors themselves acknowledge that they cannot exclude the possibility that revascularization may provide a small to moderate benefit beyond 2 years, and that they are continuing follow-up to 5 years after randomization. However, the Kaplan-Meier risk curves appear to reach a plateau within the first 2 years of follow-up, suggesting that additional follow-up may not favor revascularization (Donners et al., 2025).

Third, the sample size of ECST-2 (429 patients) was substantially smaller than that of CREST-2 (1,245 in the *stent* trial and 1,240 in the endarterectomy trial), limiting the statistical power to detect differences between groups. Fourth, only 10 of 214 patients in the revascularization group of ECST-2 received carotid *stent*, with the majority undergoing endarterectomy, which precludes direct comparisons with the *stent* trial of CREST-2 (Brott et al., 2026; Donners et al., 2025).

Finally, the published article on ECST-2 and its supplement do not clarify how adherence to OMT for achieving the proposed targets occurred. At 1 year, more than 93% and 84% of patients were reported as adherent to statin and antihypertensive therapy, respectively. It is possible that greater adherence to optimized medical therapy may account for the difference between the ECST-2 and CREST-2 studies, as the latter does not report on participant adherence (Brott et al., 2026; Donners et al., 2025).

#### 4.2.1 Methodological debate surrounding CREST-2

The methodological foundations of CREST-2 have been the subject of a recent scholarly debate that merits incorporation into the contemporary critical reading of the trial. In a 2026 commentary published in *Stroke*, Chimowitz argued that several methodological decisions may have favored the revascularization arms of CREST-2, identifying four principal concerns: (I) the exclusion of postoperative myocardial infarction as a prespecified endpoint, in contrast to CREST-1, where MI was pivotal in demonstrating equivalence between CAS and CEA—a tipping-point analysis suggested that as few as three additional MI events in the CAS arm would have eliminated its statistical advantage over OMT; (II) the absence of mandatory neurological assessment by an independent neurologist, allowing operator-team designees to perform postprocedural neurological assessments and potentially under-ascertaining mild deficits; (III) the highly selective credentialing of CAS operators (only 46% of applicants ultimately approved after additional training), which limits the external generalizability of the 1.3% periprocedural stroke-or-death rate observed in the trial; and (IV) the use of 4-year event proportions as the primary analytic method, which weighted early procedural events equally with late events occurring under medical therapy, a methodological choice that may have systematically favored revascularization under conditions of non-proportional hazards (Chimowitz, 2026).

The CREST-2 investigators (Howard, Meschia, Lal, and Brott) issued a detailed point-by-point response in the same journal, providing several important clarifications: (I) postoperative hemorrhages were prespecified as components of the primary endpoint and were systematically adjudicated, with no periprocedural hemorrhagic events in the CAS arm and three in the CEA arm; (II) the decision to exclude MI was endorsed by the NINDS peer-review process and the U.S. Food and Drug Administration, and reflects the consensus reached during the design phase that the inclusion of biomarker-confirmed MI had been a contested feature of CREST-1; (III) of the 411 suspected stroke events screened during the trial, only 190 (46%) were adjudicated as true strokes, with the proportion confirmed not differing by treatment assignment, suggesting that under-ascertainment of strokes was unlikely to have materially affected the conclusions; and (IV) the restricted mean survival time analysis was prespecified as a secondary analytic method, was included in the original manuscript submitted to *The New England Journal of Medicine*, and will be reported in subsequent analyses alongside additional weighting schemes (Howard et al., 2026).

The 2026 editorial of Hicks, Veith, and Perler (Hicks et al., 2026) further contextualizes these debates, emphasizing three pragmatic considerations for the translation of CREST-2 findings to routine clinical practice: first, the approximately 1.5% annual stroke risk achieved under intensive contemporary medical therapy is remarkable but likely to be difficult to reproduce in community settings, where adherence is imperfect and access to preventive care is variable; second, the highly selective inclusion criteria for transfemoral CAS—particularly the exclusion of patients with type III aortic arches, extensive aortic atheroma, severe ICA tortuosity or calcification, and longer lesions—render the trial population substantially less representative of everyday practice than that of CEA; and third, the conspicuous absence of TCAR from CREST-2, despite its rapid contemporary adoption in the United States, leaves an important evidentiary gap that will likely shape future trials (Hicks et al., 2026). These ongoing debates underscore the importance of nuanced, individualized, and shared decision-making in the management of asymptomatic carotid stenosis, rather than the categorical extrapolation of trial results to all clinical scenarios.

### 4.3 Comparison between endarterectomy and *carotid* stenting

Randomized clinical trials that directly compared endarterectomy and carotid *stent* in symptomatic patients (SPACE, EVA-3S, ICSS, CREST-1, and CREST-2) consistently demonstrated an excess periprocedural risk of stroke with first-generation carotid *stents*. An individual patient data meta-analysis of these trials showed an absolute increased risk of 3.2% (95% CI, 1.7%−4.7%) of periprocedural stroke or death with *stent* compared with endarterectomy, with an effect apparently modified by age (Müller et al., 2020).

In asymptomatic patients, the ACST-2 trial directly compared endarterectomy vs. *carotid* stenting (predominantly first-generation) and found no difference in the rate of death, myocardial infarction, or any stroke (3.2% CEA vs. 3.9% CAS; *p* = 0.22), although a 1% increase in the risk of non-disabling stroke associated with *stent* was observed (1.6% CEA vs. 2.7% CAS; *p* = 0.03). There was no difference in death or disabling stroke within the first 30 days (1% CEA vs. 0.9% CAS; *p* = 0.77) (Halliday et al., 2021).

A pivotal age-stratified analysis of CREST-1 published in 2011 (Voeks et al., 2011) provided a robust mechanistic basis for the heterogeneity of CAS-vs.-CEA outcomes across patient subgroups. In this prespecified analysis of 2,502 patients (1,321 symptomatic and 1,181 asymptomatic), the relative efficacy of CAS compared with CEA was significantly modified by patient age (*P* interaction = 0.02 for the primary composite endpoint; *P* interaction = 0.033 for stroke), with CAS and CEA showing equivalent risk at approximately 70 years for the composite endpoint and at approximately 64 years for the stroke endpoint. For CAS-treated patients, the hazard ratio for the primary endpoint increased by 1.77-fold per 10-year increment in age (95% CI, 1.38–2.28), whereas no significant age-related risk gradient was observed for CEA-treated patients (HR 1.16; 95% CI, 0.89–1.50; *p* = 0.27). This age effect was not modified by symptomatic status (*p* = 0.96) nor by sex (*p* = 0.45). The biological substrate proposed for this finding centers on age-related increases in aortic arch tortuosity, atheromatous burden of the aortic arch, and internal carotid artery calcification, which collectively elevate the embolic risk during transfemoral catheter navigation (Voeks et al., 2011). The subsequent individual patient data meta-analysis of SPACE, EVA-3S, and ICSS confirmed this age-related differential, with a hazard ratio of 2.04 (95% CI, 1.48–2.82) favoring CEA in patients ≥70 years (Müller et al., 2020). These findings provide a coherent rationale for the contemporary positioning of TCAR as the preferred endovascular option in older patients, since it bypasses the aortic arch in its entirety and establishes proximal cerebral protection prior to lesion crossing—addressing the two principal mechanisms hypothesized to underlie the excess periprocedural embolic risk of transfemoral CAS in elderly populations (Voeks et al., 2011; Dodwad et al., 2026; Kwolek et al., 2015).

A provocative interpretation of the CREST-2 results is that carotid *stenting* may have outperformed the surgical intervention, as it demonstrated a lower rate of periprocedural events when compared with endarterectomy in asymptomatic patients who were adequately selected and treated to achieve contemporary OMT targets. In the *stent* trial of CREST-2, the periprocedural rate of stroke or death was 1.4% (eight events in 616 patients), while in the endarterectomy trial it was 1.5% (nine events in 617 patients), essentially equivalent rates regarding participant adherence (Brott et al., 2026; Donners et al., 2025).

### 4.4 Comparison between OMT alone and *carotid* stenting

As summarized in Table 2, the randomized trials directly comparing endarterectomy and carotid stenting in symptomatic patients (SPACE, EVA-3S, ICSS, CREST-1, and CREST-2) consistently demonstrated an excess periprocedural risk of stroke with first-generation carotid stents. An equally significant consideration pertains to whether the advantages observed over the 4-year study duration justify the initial elevation of risk associated with stent placement. In the CREST-2 trial, the incidence of periprocedural stroke or mortality associated with stent placement was 1.3%, whereas no early adverse events were documented with medical therapy alone. Subsequently, the incidence of ipsilateral stroke was recorded at 0.4% per person-year in the stenting cohort, in contrast with 1.7% per person-year in the medical therapy cohort. Thus, among 100 patients who received a stent, only approximately one individual annually would experience a benefit in stroke avoidance, counterbalanced by approximately one patient who sustained an ischemic stroke or mortality resulting from the procedure. Over 4 years, 95 of every 100 patients would have undergone an unnecessary intervention (Brott et al., 2026; Brown and Bonati, 2026).

It is also pertinent to note that approximately two-thirds of the events occurring only in patients who received OMT were non-disabling strokes. Typically, these patients present with favorable or moderate recovery, subsequently requiring revascularization for the treatment of symptomatic carotid stenosis. Therefore, the immediate initiation of OMT in patients with asymptomatic carotid stenosis, with revascularization being deferred until the emergence of symptoms, which will likely manifest in only a small fraction of patients, appears to be the approach with the greatest benefit. In this context, exceptions may be justified for patients who choose to accept the risks associated with revascularization or for those unable to adhere to medical therapy, in whom stent placement appears to be the intervention of choice for appropriate patients at institutions with proficient and experienced interventionists (Brott et al., 2026; Brown and Bonati, 2026).

### 4.5 Technological evolution in carotid *stent*

Technological advances in carotid stent represent a crucial factor in the interpretation of contemporary results. First-generation *stents* employed single-layer devices with risk of plaque prolapse through the *stenting* mesh, and predominantly, distal embolic protection devices that required unprotected crossing of the lesion. Some trials did not mandate the use of embolic protection devices in their protocols (Depuydt et al., 2020).

Important technical developments include intraprocedural cerebral protection through temporary proximal flow cessation/reversal and second-generation *stents* with anti-embolic mesh (*plaque-sequestrating, mesh-covered*), which may significantly reduce plaque-related embolism during and after the procedure. A RCT comparing conventional *stent* vs. *stent* with mesh-covered stent demonstrated a significant reduction in periprocedural cerebrovascular events with the newer-generation device (Karpenko et al., 2021).

Contemporary large-scale registries have demonstrated complication rates with carotid *stenting* substantially lower than those observed in earlier clinical trials, reflecting the combination of technological advances, improved patient selection, and greater operator experience. These developments may partly explain the favorable results for *stenting* in CREST-2.

### 4.6 Position of TCAR within the therapeutic algorithm and critical comparison with CEA, tfCAS, and OMT

TCAR is not intended to displace optimal medical therapy (OMT), which remains the indisputable cornerstone of management for any patient with extracranial atherosclerotic carotid disease and is universally endorsed by contemporary Guidelines (Musialek et al., 2025; Bonati et al., 2021). Likewise, the current evidence does not support TCAR as a substitute for CEA, which retains its longstanding role as the surgical reference standard for symptomatic patients with ≥70% stenosis and for selected patients with 50%−69% stenosis. Rather, TCAR has emerged as a third therapeutic pillar that may legitimately displace tfCAS whenever an endovascular approach is judged appropriate. A recent systematic review of 16 international guidelines published since 2015 (Bierens et al., 2025) showed that two societies [the Society for Vascular Surgery (AbuRahma et al., 2022) and the Brazilian Society of Angiology and Vascular Surgery (Ristow et al., 2024)] explicitly state that, whenever CAS is indicated, TCAR should be preferred over tfCAS due to its superior safety and effectiveness profile, while the European Society for Vascular Surgery guideline (Naylor et al., 2023) regards TCAR as an equally viable alternative to tfCAS. Critically, the comparative superiority of TCAR over CEA has not been demonstrated in any randomized controlled trial (RCT), and the available comparative data, although abundant, remain essentially observational, leaving the hierarchical status of CEA conceptually intact (Straus et al., 2024; Sakowitz et al., 2025; Columbo et al., 2025).

### 4.7 TCAR limitations of current evidence and future research directions

Notwithstanding the consistency of the favorable safety signal across diverse datasets, the existing TCAR literature is subject to methodological caveats that should temper any unqualified extrapolation. First, the absence of a randomized head-to-head comparison between TCAR and CEA represents the most important evidentiary gap; the pivotal ROADSTER trials are single-arm prospective studies without internal comparators, so all comparative inferences are necessarily indirect (Kwolek et al., 2015; Jim et al., 2026; Kashyap et al., 2020). Kashyap et al. (2020) have estimated that a properly powered superiority RCT would require enrollment of more than 100,000 patients, a figure that effectively precludes its execution under conventional trial frameworks. Target-trial-emulation strategies (Sakowitz et al., 2025) and propensity-score-matched analyses (Straus et al., 2024; Malas et al., 2022) partially mitigate this limitation but cannot eliminate residual confounding by indication, because patients selected for TCAR continue to differ systematically from CEA candidates (higher age, more cardiovascular comorbidity, more anatomically unfavorable features). Second, operator and center-volume bias may inflate the externally reported safety estimates: most series originate from high-volume vascular programs and, although the TCAR learning curve is shorter than that of tfCAS, it remains non-negligible. Third, the generalization of TCAR to young symptomatic patients with non-high-risk anatomy—the group in whom CEA has shown the largest absolute benefit—is premature, given that the standard-risk cohort of ROADSTER 3 had only 9.6% symptomatic patients (Jim et al., 2026).

These limitations may be reasonably mitigated through future research focused on the following priorities: (I) prospective multicentric randomized comparisons between TCAR and CEA stratified by symptomatic status and CMS risk category, ideally with non-inferiority designs and pre-specified subgroups; (II) standardized, blinded neurological and imaging adjudication, including diffusion-weighted magnetic resonance imaging to quantify silent embolic burden; (III) inclusion of patient-reported outcomes (procedural anxiety, recovery time, return to baseline activities), currently underrepresented in the literature; (IV) long-term durability assessments beyond 5 years regarding restenosis, ipsilateral late stroke and the interaction between intensified contemporary OMT, particularly the targets advocated by the most recent dyslipidemia and hypertension guidelines (Mach et al., 2025; Patel et al., 2025; Blumenthal et al., 2026a; Rached et al., 2025; Jones et al., 2025; Brandão et al., 2025; Blumenthal et al., 2026a; van der Born et al., 2026), and revascularization benefit; and (V) prospective integration of carotid plaque-vulnerability biomarkers (intraplaque hemorrhage, lipid-rich necrotic core, ulceration, and Carotid Plaque-RADS scoring) into TCAR patient selection, given that current trials and guidelines remain anchored on stenosis severity, a recognized imperfect predictor of stroke recurrence (Dodwad et al., 2026). Until these gaps are filled, TCAR should be regarded as a technically reproducible, safe and minimally invasive complementary modality whose precise position within the contemporary therapeutic hierarchy remains only partially defined.

### 4.8 Current guidelines recommendations for carotid stenosis

The Consensus Statement on stroke risk management in carotid atherosclerotic disease, endorsed by the Stroke Council of the European Society of Cardiology (ESC), establishes specific criteria for revascularization indication in carotid stenosis that substantially align with the guidelines on revascularization and *stenting* in carotid stenosis from the European Stroke Organization (ESO) of 2021. For patients with symptomatic carotid stenosis, intervention is recommended when the degree of stenosis exceeds 50% by the NASCET method, as stenosis greater than 50% remains the most robust predictor of a new vascular event following TIA (Musialek et al., 2025).

The ESO guideline corroborates this indication but stratifies the strength of recommendation according to severity—strong for 70%−99% stenosis (high quality of evidence) and weak for 50%−69% stenosis (low quality of evidence), strongly recommending against endarterectomy for stenosis below 50% (Jones et al., 2025). For asymptomatic stenosis, both documents converge in recommending intervention when the degree of stenosis exceeds 60%, provided that the patient's life expectancy is at least 2–5 years and the patient is considered at increased risk of stroke under optimal medical therapy alone (Musialek et al., 2025; Bonati et al., 2021).

Regarding procedural safety thresholds, there is agreement among societies in recognizing that traditional thresholds for the combined rate of periprocedural stroke or death (≤6% for symptomatic and ≤ 3% for asymptomatic patients) may no longer be applicable given advances in revascularization techniques. The ESC consensus states that, for both endarterectomy and *stent*, the combined periprocedural rate of stroke or death should not exceed 2% for asymptomatic stenoses and 4% for symptomatic stenoses (Musialek et al., 2025), values identical to those established by the ESO guideline, stating that the in-hospital risk should be as low as possible, ideally below 2% for asymptomatic patients and should not exceed 4% for symptomatic patients (Bonati et al., 2021). These more restrictive thresholds represent a revision of the traditional values of 3% and 6%, respectively, reflecting technical evolution and the need for a more robust net benefit against contemporary optimal medical therapy.

Beyond the degree of stenosis, both documents emphasize that carotid plaque morphology and composition play an important role in risk stratification and contemporary clinical decision-making. The ESO highlights as markers of increased risk silent infarction on neuroimaging, stenosis progression, echolucent plaque on ultrasound, intraplaque hemorrhage on magnetic resonance imaging, and microembolic signals on transcranial Doppler (Bonati et al., 2021). The ESC consensus broadens this approach, incorporating additional plaque vulnerability characteristics: increased volume, neovascularization, ulcerations, endothelial erosions, extensive lipid-rich necrotic cores, and thin or ruptured fibrous cap, with recent data demonstrating that the latter is associated with a higher stroke risk than stenosis severity alone (Musialek et al., 2025).

The ESC publication further recommends that assessment of stenosis severity by CTA or MRA be performed before any intervention, since Doppler, although the first-line examination for stenosis identification, has limited accuracy in the precise determination of moderate-to-severe stenosis grades (Musialek et al., 2025). Documented stenosis progression and the presence of silent cerebral infarctions on neuroimaging should also be considered in therapeutic decision-making (Musialek et al., 2025; Bonati et al., 2021).

The 2023 European Society for Vascular Surgery (ESVS) Clinical Practice Guidelines on the Management of Atherosclerotic Carotid and Vertebral Artery Disease (AbuRahma et al., 2022) formally incorporate the concept of high-risk plaque features into the indication algorithm for revascularization, both in symptomatic and in asymptomatic patients. For average surgical risk patients with 60%−99% asymptomatic stenosis, the ESVS recommends consideration of carotid endarterectomy (Class IIa, Level of Evidence B) and may consider CAS (Class IIb, Level of Evidence B) in the presence of one or more of the following imaging or clinical characteristics associated with an increased risk of late stroke under OMT alone: silent ipsilateral infarction on cerebral CT or MRI; stenosis progression by ≥20%; intraplaque hemorrhage on MRI; impaired cerebrovascular reserve; ≥1 spontaneous microembolic signal during ≥1 h of transcranial Doppler monitoring; large juxta-luminal hypoechoic (black) area on computerized plaque analysis (≥8 to 10 mm^2^); and predominantly echolucent plaque on duplex ultrasound—provided that the 30-day procedural stroke or death rate is documented as < 3% and the patient's life expectancy exceeds 5 years (Naylor et al., 2023; Paraskevas and AbuRahma, 2024). This recommendation is supported by a meta-analysis (Hackam, 2021) of 64 studies (*n* = 20,751 asymptomatic patients with carotid stenosis), which reported that approximately 26.5% of these patients exhibit at least one high-risk plaque feature and that the presence of such features is associated with a three-fold increase in ipsilateral ischemic cerebrovascular events (annual rate of 4.3% vs. 1.2%; OR 3.0; 95% CI, 2.1–4.3; *p* < 0.001), with intraplaque hemorrhage demonstrating the strongest individual association (OR 7.0) (Hackam, 2021).

This plaque-and-patient-based approach increasingly complements and contrasts with the predominantly stenosis-centric framework that has historically governed therapeutic decision-making. The 2024 comparative analysis of Paraskevas and AbuRahma (2024) identifies the formal incorporation of plaque vulnerability criteria as one of the principal discriminating features between the 2023 ESVS guidelines and the 2022 Society for Vascular Surgery (SVS) guidelines, the latter of which adopts a more stenosis-focused approach but similarly endorses TCAR as the preferred endovascular option in patients with ≥50% symptomatic stenosis when an endovascular approach is judged appropriate (Paraskevas and AbuRahma, 2024). For symptomatic patients, the ESVS Class I recommendation for revascularization within 14 days of the index event applies regardless of plaque morphology when stenosis is ≥70%, although plaque vulnerability features may further support intervention in selected patients with moderate (50%−69%) stenosis. The progressive integration of plaque imaging (particularly vessel-wall MRI with detection of intraplaque hemorrhage) into clinical decision algorithms represents one of the most promising avenues for individualized risk stratification in the coming decade, and constitutes a central pre-specified element in the design of the next generation of carotid revascularization trials (Paraskevas and AbuRahma, 2024; Bierens et al., 2025; Hackam, 2021).

### 4.9 Current recommendations from other guidelines

Other recently published documents on the management of blood pressure and dyslipidemias in general contexts, not specific to patients with carotid stenosis, stipulate more stringent targets than those mentioned above.

The 2024 European Hypertension Guideline states that the target for adults diagnosed with hypertension should be an SBP between 120 and 129 mmHg and a DBP below 80 mmHg, provided that treatment is well-tolerated. For frail elderly patients presenting with signs of arterial hypotension or who have a short life expectancy, more lenient targets (“as low as possible, based on tolerability”) are considered acceptable (Blumenthal et al., 2026a; van der Born et al., 2026). The 2025 American Guideline establishes a target for adults with confirmed hypertension as blood pressure below 130/80 mmHg, with an incentive for further systolic reduction to below 120 mmHg when feasible and safe (Donners et al., 2025). The 2025 Brazilian Guideline aligns with global standards—primarily European ones—and suggests a target for most adults with hypertension of blood pressure below 130/80 mmHg, while allowing more permissive targets for frail elderly patients or those at high risk of adverse outcomes, such as falls due to postural hypotension (Brandão et al., 2025).

A point of convergence among these guidelines is the recommendation that treatment be adapted in situations of intolerance, presence of other health conditions, or frailty. They reflect the current understanding that achieving lower blood pressure targets is associated with a greater reduction in cardiovascular risk, provided that treatment is individualized and well-tolerated (Jones et al., 2025; Brandão et al., 2025; Blumenthal et al., 2026a; van der Born et al., 2026).

Regarding LDL-c targets, the 2025 update of the European Dyslipidemia Management Guidelines, the 2025 American Association of Clinical Endocrinology Guidelines, and the 2026 American College of Cardiology/American Heart Association Guidelines all recommend an LDL-c target < 55 mg/dL for patients at very high cardiovascular risk or with established atherosclerotic disease (Rached et al., 2025; Rodrigues et al., 2025; Hackam, 2021). In contrast, the Brazilian Guideline establishes an LDL-c target of < 50 mg/dL for those at very high cardiovascular risk, classifying patients with carotid stenosis greater than 50% of the lumen as members of this group (Rached et al., 2025).

Although not directed at patients with carotid stenosis, these trends toward more stringent risk factor control, achievable owing to the evolution of pharmacological therapies, raise questions regarding how they would impact the decision to indicate or not endarterectomy or angioplasty with *stent* for these individuals.

### 4.10 Practical clinical implications

The findings of this review carry important practical clinical implications. First, OMT remains the fundamental pillar of management for all patients with carotid stenosis, regardless of the decision regarding revascularization. All patients must cease smoking, receive antiplatelet and statin therapy, with stringent control of LDL-c levels and blood pressure, in addition to lifestyle modification interventions.

Patient selection for revascularization should be individualized, considering not only the degree of stenosis but also plaque characteristics, symptoms, life expectancy, comorbidities, and patient preferences. The CAR score used in ECST-2 represents an advance in risk stratification, although it requires additional validation in different populations.

Moreover, the CREST-2 results suggest that carotid *stenting* may be a preferred option in selected asymptomatic patients, particularly when performed by experienced operators using contemporary technology. The absence of significant benefit of endarterectomy over optimal medical therapy in CREST-2 demands critical reassessment of the role of this procedure in asymptomatic patients.

An equally significant consideration pertains to whether the advantages observed over the 4-year study duration justify the initial elevation of risk associated with stent placement. In the CREST-2 trial, the incidence of periprocedural stroke or mortality associated with stent placement was 1.3%, whereas no early adverse events were documented with medical therapy alone. Subsequently, the incidence of ipsilateral stroke was recorded at 0.4% per person-year in the stenting cohort, in contrast with 1.7% per person-year in the medical therapy cohort. Thus, among 100 patients who received a stent, only approximately one individual annually would experience a benefit in stroke avoidance, counterbalanced by approximately one patient who sustained an ischemic stroke or mortality resulting from the procedure. Over 4 years, 95 of every 100 patients would have undergone an unnecessary intervention (Brott et al., 2026; Brown and Bonati, 2026).

It is also pertinent to note that approximately two-thirds of the events occurring only in patients who received OMT were non-disabling strokes. Typically, these patients present with favorable or moderate recovery, subsequently requiring revascularization for the treatment of symptomatic carotid stenosis. Therefore, the immediate initiation of OMT in patients with asymptomatic carotid stenosis, with revascularization being deferred until the emergence of symptoms, which will likely manifest in only a small fraction of patients, appears to be the approach with the greatest benefit. In this context, exceptions may be justified for patients who choose to accept the risks associated with revascularization or for those unable to adhere to medical therapy, in whom stent placement appears to be the intervention of choice for appropriate patients at institutions with proficient and experienced interventionists (Brott et al., 2026; Brown and Bonati, 2026).

Last but not least, shared decision-making with the patient assumes increasing relevance, given that the currently available evidence does not establish a single clearly superior strategy for all patients with asymptomatic carotid stenosis.

#### 4.10.1 Timing of carotid revascularization

The timing of carotid revascularization following the index symptomatic event constitutes one of the most consistent and powerful determinants of clinical benefit identified in the contemporary literature. The pooled analysis by Rothwell et al. of NASCET and ECST individual patient data demonstrated that the absolute benefit of carotid endarterectomy in patients with ≥ 50% symptomatic stenosis is maximal when surgery is performed within 14 days of the index event, with the number needed to treat to prevent one ipsilateral stroke at 5 years rising progressively from approximately 5 (intervention within 2 weeks) to 125 (intervention beyond 12 weeks). This finding reflects the dynamic nature of early plaque instability following a sentinel cerebrovascular event, during which the risk of recurrent ipsilateral stroke is concentrated in the first weeks (Rothwell et al., 2004).

Both the 2022 SVS and the 2023 ESVS clinical practice guidelines endorse revascularization within 14 days of symptom onset in patients deemed appropriate for the procedure [Grade 1 (Strong), Quality of Evidence B in SVS; Class I, Level of Evidence A in ESVS] (Naylor et al., 2023; AbuRahma et al., 2022). Both documents simultaneously issue strong recommendations against acute intervention in patients with disabling stroke, infarction exceeding 30% of the ipsilateral middle cerebral artery territory, modified Rankin Scale score ≥3, or altered consciousness, due to the increased risk of postoperative parenchymal hemorrhage in these patients, who may be re-evaluated for revascularization if neurological recovery proves satisfactory (Paraskevas and AbuRahma, 2024). Among patients undergoing revascularization within the first 14 days following symptom onset, both guidelines specifically recommend carotid endarterectomy over transfemoral carotid stenting [Grade 1 (strong) in SVS; Class I, Level of Evidence A in ESVS], reflecting the consistent finding across SPACE, EVA-3S, ICSS, and CREST-1 that early CAS is associated with disproportionately higher periprocedural stroke risk compared with CEA, particularly in patients above 70 years of age (Voeks et al., 2011; Paraskevas and AbuRahma, 2024; Müller et al., 2020). For symptomatic patients, the timely identification of the index event (supported by ABCD Feigin et al., 2025 score risk stratification and prompt vascular Imaging) therefore constitutes a critical step in the operational chain of care that defines the magnitude of benefit attainable through revascularization.

### 4.11 Conclusion

This review on the treatment of carotid stenosis allows the following conclusions to be drawn:

The treatment of atherosclerotic carotid stenosis is at a moment of paradigm shift. The CREST-2 results (Paraskevas and AbuRahma, 2024) suggest that the addition of carotid *stent* to optimal medical therapy significantly reduced the risk of the composite primary endpoint (stroke or death periprocedural and ipsilateral stroke at 4 years) compared with OMT in patients with high-grade asymptomatic stenosis. However, there were more periprocedural deaths and strokes with stenting compared with OMT alone (Brott et al., 2026).

Surprisingly, carotid endarterectomy, a procedure considered the gold standard for over three decades based on the ACAS and ACST-1 trials, did not demonstrate statistically significant benefit over OMT alone in CREST-2 (Brott et al., 2026). This finding challenges established concepts and demands critical reassessment of the indications for endarterectomy in asymptomatic patients.

As CREST-2 contains two parallel *trials*, we remain methodologically limited in comparing endarterectomy vs. *stent*. Perhaps a future study with three arms (control, endarterectomy, and *stent*) may be able to more precisely answer which of the two interventional methods is superior.

The ECST-2 trial (Brott et al., 2026), in its 2-year interim analysis, did not demonstrate benefit of revascularization added to optimal medical therapy in patients with asymptomatic or low-to-intermediate-risk symptomatic stenosis. However, substantial methodological differences between ECST-2 and CREST-2, including selection criteria, follow-up duration, and sample size, limit direct comparisons between the studies.

OMT remains the most important pillar of management for all patients with carotid atherosclerotic disease. Pharmacological advances, particularly the intensive use of statins with stringent LDL-c targets, have substantially reduced the baseline risk of events but have not completely eliminated the risk of stroke in patients with significant stenosis.

New studies are needed evaluating, in patients with carotid stenosis, the impact of achieving the currently recommended blood pressure and lipid targets. Future comparative analyses are also needed between these targets alone vs. in combination with endarterectomy or angioplasty.

Individualized risk stratification, incorporating not only the degree of stenosis but also morphological plaque characteristics, time since the index event, comorbidities, and life expectancy, remains fundamental for appropriate therapeutic decisions. Tools such as the CAR score represent promising advances in this direction.

#### 4.11.1 Limitations

This review has limitations inherent to its narrative design, which does not employ systematic search and study selection methodology. The methodological heterogeneity among the analyzed trials hinders direct comparisons and quantitative syntheses. Additionally, the results of the most recent studies reflect specific contexts of medical treatment, procedural techniques, and patient selection that may not be directly generalizable to all clinical settings.

#### 4.11.2 Future perspectives

Important knowledge gaps remain and require investigation in future research. These include: prospective validation of risk stratification models; direct comparison of second-generation carotid stent vs. contemporary endarterectomy; the role of biomarkers and plaque imaging characteristics in patient selection; comparison of OMT with current more stringent targets alone vs. combined with endarterectomy or angioplasty; efficacy of novel pharmacological agents in event prevention; and long-term follow-up (exceeding 4–5 years) of the different therapeutic strategies.
